# Par system components are asymmetrically localized in ectodermal epithelia, but not during early development in the sea anemone *Nematostella vectensis*

**DOI:** 10.1186/s13227-015-0014-6

**Published:** 2015-05-09

**Authors:** Miguel Salinas-Saavedra, Thomas Q Stephenson, Casey W Dunn, Mark Q Martindale

**Affiliations:** The Whitney Laboratory for Marine Bioscience, University of Florida, 9505 N, Ocean Shore Blvd, St. Augustine, FL 32080-8610 USA; Department of Ecology and Evolutionary Biology, Brown University, Providence, RI 02912 USA

**Keywords:** Cnidaria, Bilateria, Polarity, *Nematostella vectensis*, Par proteins

## Abstract

**Background:**

The evolutionary origins of cell polarity in metazoan embryos are unclear. In most bilaterian animals, embryonic and cell polarity are set up during embryogenesis with the same molecules being utilized to regulate tissue polarity at different life stages. Atypical protein kinase C (aPKC), lethal giant larvae (Lgl), and *Par*titioning-defective (Par) proteins are conserved components of cellular polarization, and their role in establishing embryonic asymmetry and tissue polarity have been widely studied in model bilaterian groups. However, the deployment and role of these proteins in animals outside Bilateria has not been studied. We address this by characterizing the localization of different components of the Par system during early development of the sea anemone *Nematostella vectensis*, a member of the clade Cnidaria, the sister group to bilaterian animals.

**Results:**

Immunostaining using specific *N. vectensis* antibodies and the overexpression of mRNA-reporter constructs show that components of the *N. vectensis* Par system (*Nv*Par-1, *Nv*Par-3, *Nv*Par-6, *Nv*aPKC, and *Nv*Lgl) distribute throughout the microtubule cytoskeleton of eggs and early embryos without clear polarization along any embryonic axis. However, they become asymmetrically distributed at later stages, when the embryo forms an ectodermal epithelial layer. *Nv*Lgl and *Nv*Par-1 localize in the basolateral cortex, and *Nv*aPKC, *Nv*Par-6, and *Nv*Par-3 at the apical zone of the cell in a manner seen in bilaterian animals.

**Conclusions:**

The cnidarian *N. vectensis* exhibits clear polarity at all stages of early embryonic development, which appears to be established independent of the Par system reported in many bilaterian embryos. However, in *N. vectensis*, using multiple immunohistochemical and fluorescently labeled markers *in vivo*, components of this system are deployed to organize epithelial cell polarity at later stages of development. This suggests that Par system proteins were co-opted to organize early embryonic cell polarity at the base of the Bilateria and that, therefore, different molecular mechanisms operate in early cnidarian embryogenesis.

**Electronic supplementary material:**

The online version of this article (doi:10.1186/s13227-015-0014-6) contains supplementary material, which is available to authorized users.

## Background

**Figure 1:**
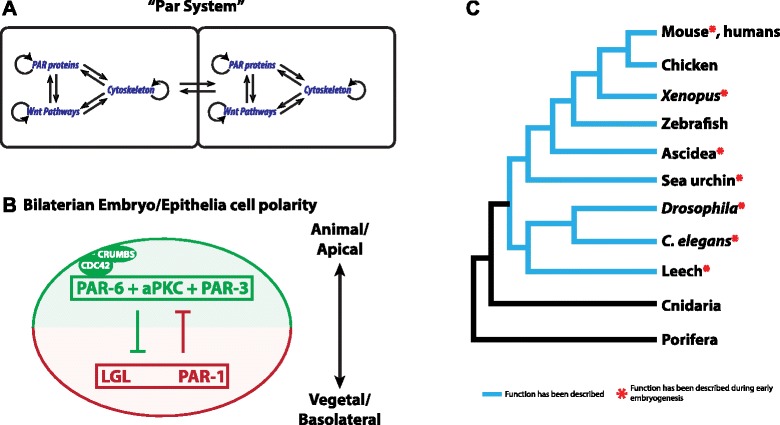
Components of the Par system are conserved across metazoans. **(A)** The ‘Par system’ is a set of regulatory proteins that direct the polarity of the cell. For the examined bilaterian animals, the Par system is influenced by the interaction between cell-cell junctions, cytoskeletal elements, and components of the Wnt signaling pathways. **(B)** In bilaterian animals, Par-3, Par-6, and aPKC form a bi/tripartite complex that localizes to apical regions of the cell, binding to CDC42 and CRUMBS. In contrast, both Lgl and Par-1 localize at the basolateral cortex of the cell. Mutual antagonism by phosphorylation has been proposed as the mechanism that controls the segregation of these two distinct cortical domains. **(C)** Par proteins are present in the genome of sequenced Metazoa, including Cnidaria, Ctenophores, Porifera, and Placozoa. However, the function of Par proteins, in epithelial (blue branches) and early embryogenesis (red asterisk), has only been described for some bilaterian animals, and there are no descriptions available for non-bilaterian animals (black branches).

Virtually, all cells in multicellular animals are structurally and functionally polarized. Crawling mesenchymal cells have a leading edge, and epithelial tissues have an apical domain and a basal lateral domain [1,2]. Polarity in epithelial tissues is known to be influenced by cell-cell junctions, cytoskeletal elements, and by cell-cell signaling such as components of Wnt signaling pathways [3-11] that all converge on a set of regulatory proteins referred to in this paper as the ‘Par system’ (Figure 1A). The basic mechanism by which Par proteins generate polarity cues in a cell is directed by a set of five principal proteins: Par-1, Par-3, Par-6, atypical protein kinase C (aPKC), and lethal giant larvae (Lgl). All these proteins constitutively localize at the cell cortex and migrate to the cytoplasm when they are inactivated by phosphorylation. Par-3, Par-6, and aPKC form a bi/tripartite complex that localizes to different regions of the apical cortex of the cell, binding to the activated Rho protein CDC42 and CRUMBS (Figure 1B) [1,2,12-14]. In contrast, both Lgl and Par-1 localize at the basolateral cortex of the cell (Figure 1B) [1,2,15], but it is unclear whether these two components act individually or as an associated complex. The segregation and maintenance of these two distinct cortical domains appears to be achieved through mutual antagonism. Par-1 and Lgl are inactivated and excluded from the apical cortex when they are phosphorylated by aPKC, which is activated at the apical cortex [11]. Likewise, the Par-3/Par-6/aPKC complex is excluded from the basolateral cortex following the phosphorylation of Par-3 by Par-1 [11]. This general mechanism is conserved in all bilaterian animals that have been studied (Figure 1C) [3,10,11,16-19].

Embryonic cell polarity arises during early development and is critical for axial organization (for example, the oral-aboral, the anterior-posterior, or the dorso-ventral axis). In most bilaterian animals, the oocyte already possesses an internal polarity that is set up maternally called the animal-vegetal axis (A/V axis). Rearrangements of the egg’s cytoplasm and cortical domains in different embryos further polarize the embryo, ensuring proper partitioning of maternal determinants into distinct daughter cells during the cleavage program. The A/V polarity of the embryo and the apico-basal (A/B) polarity of epithelial tissue, in the bilaterian animals examined (including vertebrates, nematode worms, and insects; Figure 1C), have been described as the products of the interaction between the same set of Par system proteins [4-8,11,20-29].

How this complex pattern of molecular interactions arose in animal evolution is unclear. In particular, it is not yet known whether the Par system first served to polarize cells within tissues and was later co-opted to polarize embryos, or, evolved to polarize embryos, and then maintained to polarize cells/tissues during later development. The resolution of this question requires the study of non-bilaterians like cnidarians. Cnidarians (sea anemones, corals, hydroids, and ‘jellyfish’) are composed of polarized outer (epidermal) and inner (gastrodermal) tissue and are the sister group to the group of Bilateria. This phylogenetic position makes them pivotal for reconstructing the evolutionary history of the Par system. Interest in the molecular mechanisms regulating cnidarian development has grown significantly in the past decade, due, in part, to the existence of genomic sequence information. However, while there are some studies that have focused on Wnt signaling pathways [30-35], cytoskeleton [36,37], and cell junctions [38], there is no published data about the localization and function of Par proteins relative to embryonic and tissue cell polarity in cnidarians. In addition, cnidarians undergo gastrulation at the animal pole of the egg, not the vegetal pole like bilaterian embryos [39-43], so the establishment of embryonic polarity in cnidarians is even more interesting. Par proteins are highly conserved proteins in the genome of Metazoa (including Cnidaria) (Figure 1C) [44], which raises the question: are the same molecular mechanisms conserved for specifying A/V and epithelial cell polarity in Cnidaria as in Bilateria?

Functional disruption of the Par system in bilaterian embryos leads to failures in the segregation of maternal components and disruptions in the orientation of cell divisions, directly affecting the polarity of the cell [28,29,45,46]. Interestingly, under normal conditions, the early embryos of the sea anemone, *Nematostella vectensis*, show similar characteristics to Par system mutants of some bilaterian animals. The early embryos of *N. vectensis* develop with a series of random, asymmetric, and non-synchronous cleavages without clear polarity [32,47]. They possess weak cell junctions, and it is practically impossible to determine the number of cells or cell cycles without any markers due to the lack of characteristic cell boundaries (Additional file 1). In spite of this ‘chaotic cleavage program,’ *N. vectensis* embryos successfully give rise to an organized and polarized monolayer of blastula epithelia on their way to polyp formation.

Are Par system proteins localized in *N. vectensis,* and if so, when? Does their distribution suggest that the Par system plays a role in embryonic polarity, tissue polarity, or both? This study attempts to address these questions by giving a general characterization of the most common proteins of the Par system during the early development of the starlet sea anemone *N. vectensis*. Results reported here will provide a framework for future functional experiments to get a better understanding on the evolution of cell polarity in Metazoa.

## Methods

### Embryo collection

Spawning, gamete preparation, fertilization, and embryo culturing were performed as previously described [48-50].

#### *In situ* hybridization

*In situ* hybridization was carried out following a previously published protocol for *N. vectensis* [51]. Animals were fixed in ice-cold 4% paraformaldehyde with 0.2% glutaraldehyde in 1/3× seawater for 2 min, followed by 4% paraformaldehyde in PBTw for 1 h at 4°C. Digoxigenin (DIG)-labeled probes, ranging from 449 to 1,770 base pairs, were hybridized between 65°C and 70°C for 2 days and developed with the enzymatic reaction of NBT/BCIP as substrate for the alkaline phosphatase-conjugated anti-DIG antibody (Cat.#11093274910; Roche, Inc., Nutley, NJ, USA). Samples were developed until gene expression was visible as a purple precipitate. The cloned genes utilized are listed in Table 1.

**Table 1:** **List of primer sequences used to clone the genes used in this study**

**Gene**	**Accession number**	**Forward primer**	**Reverse primer**
*Nv*aPKC (full length)	XP_001637001	5′-ATGATGAATTCTACGAGTGCAA	5′-GACTGAGTCTTCTACTGACATA
*Nv*Par-6 (full length)	XP_001627416	5′-ATGTCGAAGCTACAAAAGCAGT	5′-TATTGATAGAATACCAGTCTCA
*Nv*Par-3 (full length)	XP_001637950	5′-ATGATGAAGGTTGTAGT	5′-CACGCGTGTAGGCTGTGATA
*Nv*Par-3 (probe)	XP_001637950	5′-CGAGAGCAAGGGAAACAGGT	5′-GATGTAGGAGGCTCGACACG
*Nv*Lgl (full length)	XP_001640099	5′-ATGTTCAAGTTCTTGCATAGAG	5′-CAATTCTGAAGCAGTCAGAGAG
*Nv*Lgl (probe)	XP_001640099	5′-TTGAGCTGCAGGTGACAGAG	5′-AGTTTCGTCGACTCGGCTTT
*Nv*Par-1 (probe)	XP_001622868	5′-AATATAAACTATGAACTTAACG	5′-TTAAAGTTTTAATTCATTTGCA

### Immunohistochemistry

Embryos of different stages were fixed at room temperature in fresh 3.4% formaldehyde, HEPES 0.1 M (pH 6.9), EGTA 0.05 M (pH 8 to 9), MgSO4 0.005 M, NaCl 0.2 M, glutaraldehyde 0.2%, Triton X-100 0.2%, phosphate-buffered saline (PBS) 1×, and pure water for 1 to 2 h at room temperature (von Dassow fixative). Fixed embryos were rinsed 5× in PBTB (PBS buffer plus 1% BSA and 0.1% Triton X-100). To visualize F-actin, embryos were incubated in Biodipy-FL phallacidin (Life Technologies, Carlsbad, CA, USA) diluted 1:200 in PBTB. Tubulin was visualized by incubation in anti-alpha tubulin (Sigma T9026, Sigma-Aldrich, St. Louis, MO, USA). Affinity-purified *N. vectensis* anti-aPKC (anti-*Nv*aPKC) and anti-Lgl (anti-*Nv*Lgl) peptide antibodies were raised against a selected amino acid region of the *Nv*aPKC protein (PTNEDLGPKRKP; Bethyl Inc., Montgomery, TX, USA) and *Nv*Lgl protein (GNFDPFSDDPR; Bethyl Inc., Montgomery, TX, USA), respectively. Affinity-purified *N. vectensis* anti-Par-1 (anti-*Nv*Par1) and anti-Par-6 (anti-Par6) peptide antibodies were previously raised by the same company (Bethyl Inc., Montgomery, TX, USA) and were kept under storage conditions in the lab. The selected amino acid regions used for *Nv*Par-1 and *Nv*Par-6 protein were CRSTFHSGERPRDRQRDE and CENPTVDNETGILSI, respectively. Blast searches against the *N. vectensis* genome sequences showed that the amino acid sequences were not present in any predicted *N. vectensis* proteins other than the expected protein. Primary antibody incubations 1:100 were carried out in blocking buffer (5% normal goat serum in PBTB) at 4°C overnight. After incubation with the primary antibodies, animals were washed with PBTB (5×) for 10 min each wash. Secondary antibodies (Alexa 594-conjugated anti-mouse and Alexa 647-conjugated anti-Rabbit, Invitrogen A21203 and A21245 (Invitrogen, Grand Island, NY, USA), respectively) were used at 1:500 to allow for visualization. All incubations were conducted over night at 4°C. Stained embryos were rinsed again in PBS (5×) and dehydrated into isopropanol using the gradient 50%, 75%, 90%, and 100% and mounted in Murray’s mounting media (MMM; 1:2 benzyl benzoate:benzyl alcohol) for visualization. We scored more than 1,000 embryos per each antibody staining and confocal imaged more than 50 embryos at each stage.

### Western blot

Western blots were carried out as described [52-55]. Antibody concentrations for Western blot were 1:2,000 for all antibodies tested.

### Microinjections

The coding region for each gene of interest was PCR-amplified. The PCR product was then cloned into pSPE3-mVenus or pSPE3-mCherry using the Gateway system [56]. Eggs were injected directly after fertilization as previously described [48,57] with the mRNA encoding one or more Par complex proteins fused in frame with reporter fluorescent proteins (*N*-terminal tag) using final concentrations between 400 and 750 ng/μl. Live embryos were kept at 16°C and visualized after the mRNA of the FP was translated into protein. Par proteins mRNAs were co-injected with *Lifeact::mTurquoise2* (*Lifeact::mTq2*) mRNA to visualize the cortex of the cells. We injected and recorded more than 500 embryos for each experiment and confocal imaged approximately 10 specimens for each stage. Live embryos were mounted in one-third sea water for visualization. Images were documented at cleavage stages (3 to 4 hpf and 9 to 10 hpf), blastula (12 to 15 hpf), and gastrula (24 to 30 hpf) stages. The cloned genes are listed in Table 1.

### Imaging of *N. vectensis* embryos

Images of live and fixed embryos were taken using a confocal Zeiss LSM 710 microscope (Carl Zeiss SMT Inc., Peabody, MA, USA) using a Zeiss C-Apochromat 40× water immersion objective (N.A. 1.20). Pinhole settings varied between 1.2 and 1.6 A.U. pinhole. z-stack images were processed using Imaris 7.6.4 (Bitplane Co., Belfast, UK) software for three-dimensional reconstructions and ImageJ for single slice and movies. Final figures were assembled using Adobe Illustrator and Adobe Photoshop (Adobe Systems, Mountain View, CA, USA).

## Results

### Identification of maternal and zygotic mRNA distribution by whole-mount *in situ* hybridization

**Figure 2:**
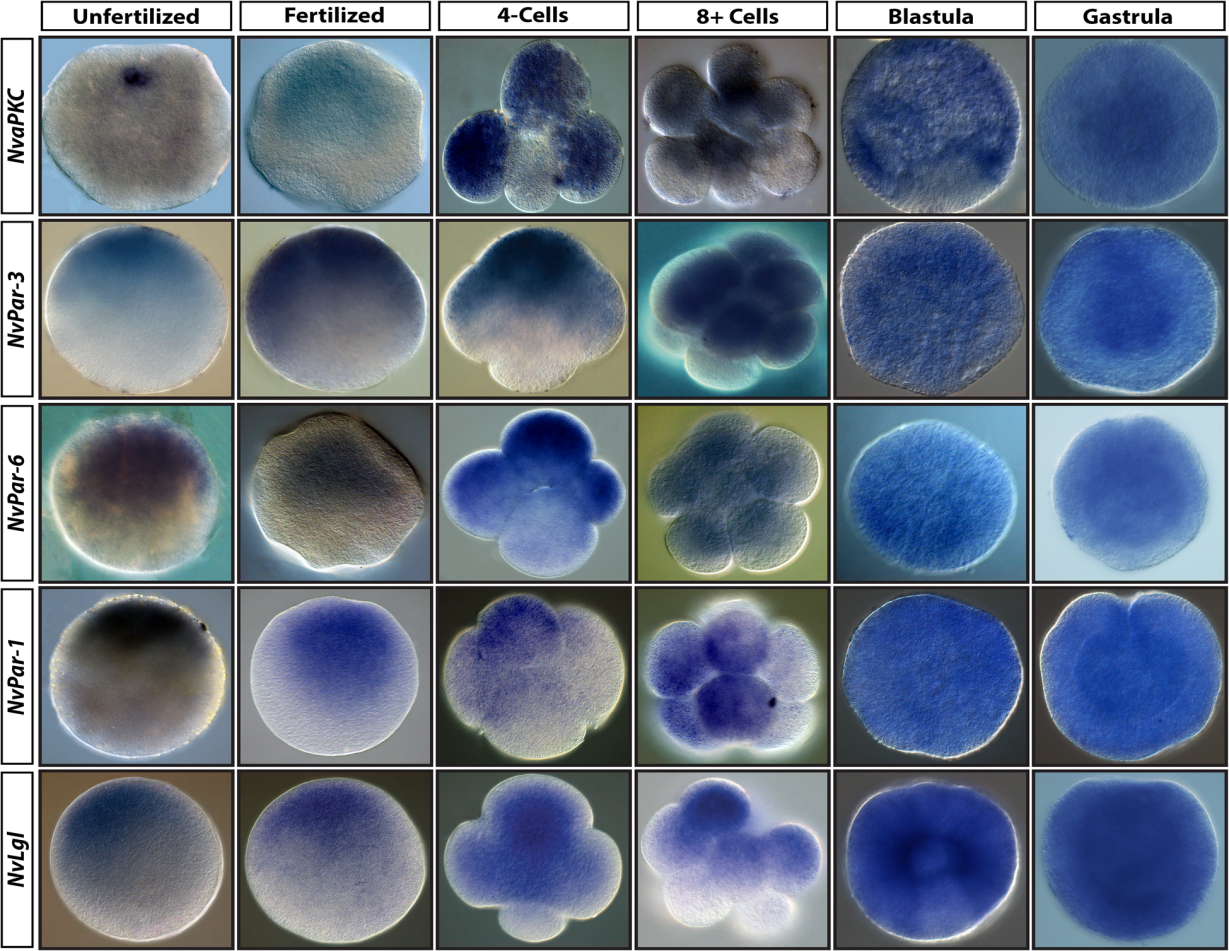
Identification of mRNA distribution by whole-mount *in sit*u hybridization. Maternal mRNA of *Nv*aPKC, *Nv*Par-3, *Nv*Par-6, *Nv*Par-1, and *Nv*Lgl are asymmetrically distributed during the early development of *N. vectensis*. In blastula and gastrula stages, these genes are uniformly distributed in the ectoderm. The distribution of all five genes is not consistent during early stages, suggesting no association with the animal-vegetal axis of the embryo; therefore, the protein localization is required.

We performed *in situ* hybridization experiments on five Par system genes (*NvaPKC, NvPar-3, NvPar-6, NvPar-1, and NvLgl*) in order to assess the distribution of the maternal and zygotic mRNA transcripts for different proteins at early stages of *N. vectensis* development (Figure 2). Transcripts of all five genes are asymmetrically distributed in unfertilized eggs, fertilized eggs, and through the early cleavage stages due to the presence of the yolk gradient (Figure 2). However, this asymmetric distribution does not appear to be correlated with A/V polarity or the subsequent cleavage program *per se* because different patterns were observed across a number of embryos at the four-cell stage (Additional file 2), suggesting that mRNA distribution in *N. vectensis* is not linked to mitotic spindle orientation and the patterns of the initial cleavages. In addition, zygotic transcripts of all five genes are uniformly distributed during blastula stage (Figure 2) and in ectodermal tissue of gastrula (Figure 2) and planula stages (data not shown). Therefore, the distribution of *NvaPKC, NvPar-3, NvPar-6, NvPar-1, and NvLgl* mRNA does not appear to be related to embryonic axis orientation.

### Antibody specificity

**Figure 3:**
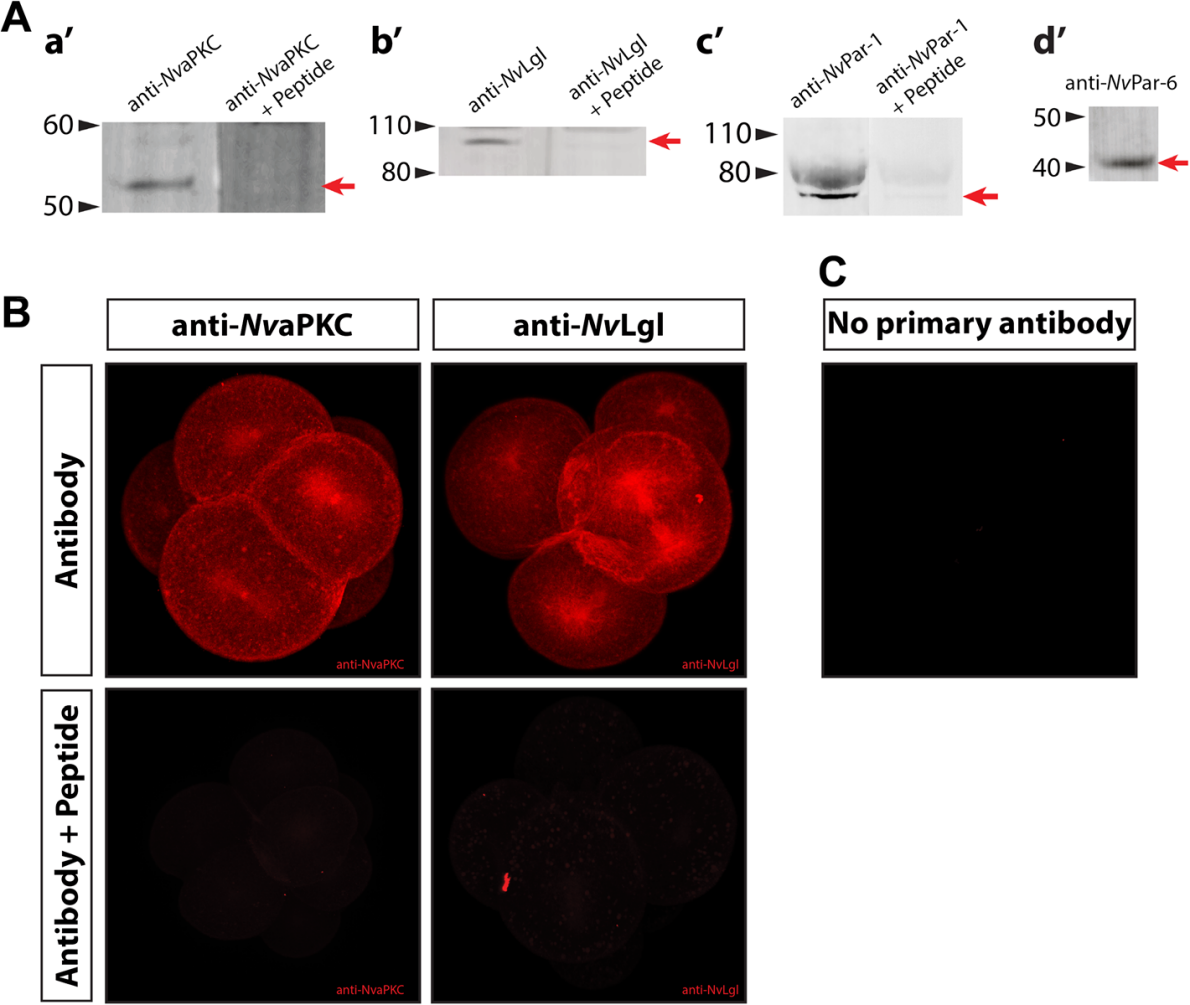
Specificity of *N. vectensis* antibodies tested by pre-adsorption experiments. **(A)** Western blots of *N. vectensis* gastrula extracts using specific antibodies against *Nv*aPKC (a′), *Nv*Lgl (b′), *Nv*Par-1 (c′), and *Nv*Par-6 (d′). Pre-adsorption of the antibodies with a tenfold excess of the antigen peptide resulted in the elimination of the staining of a single band for *Nv*aPKC and *Nv*Lgl (a′ and b′, respectively) and the titration of anti-*Nv*Par-1(c′). Arrowheads indicate the molecular weight in KD. A red arrow indicates the band recognized by the antibody for each protein. **(B)** Whole-mount immunohistochemistry pre-adsorption experiments show that the staining pattern was eliminated in early embryos when pre-incubated antibodies against *Nv*aPKC and *Nv*Lgl with the respective peptide. **(C)** shows the negative control when the primary antibody was not added.

Polyclonal affinity-purified antibodies against *N. vectensis* proteins *Nv*Lgl, *Nv*aPKC, *Nv*Par-6, and *Nv*Par-1 were used to determine the spatial and temporal expression of the protein at different stages of development. Each antibody was characterized by Western blotting to establish its specificity (Figure 3A). Western blots of *N. vectensis* gastrula extracts showed that the antibodies recognized different bands (Additional file 3) for *Nv*aPKC (predicted size 64 KD; Additional file 3A) and *Nv*Lgl (predicted size 102 KD; Additional file 3B). Pre-adsorption of the antibodies with a tenfold molar excess of the antigenic peptide (used to generate and affinity purify the antibodies) resulted in the elimination of the appropriate-sized single band for *Nv*aPKC and *Nv*Lgl (Figures 3a′,b′, respectively). Curiously, *Nv*aPKC was detected around 10 KD lower than its predicted size (Figure 3a′), perhaps as a result of posttranslational modifications. Further experiments are necessary to assess the biological meaning of this result. Anti-*Nv*Par-6 and Anti-*Nv*Par-1 antibodies showed a higher specificity by Western blot analysis. We observed two strong bands around 80 KD for *Nv*Par-1 (predicted size 86 KD; Figure 3c′) and two single bands for *Nv*Par-6 around 40 and 60 KD, respectively (predicted size 42 KD; Figure 3d′ and Additional file 3D). Pre-adsorption of the Anti-*Nv*Par-1 antibody with a tenfold excess of the antigenic peptide resulted in the titration of the stained bands (Figure 3c′). Unfortunately, we did not have the peptide for *Nv*Par-6 antibody to perform pre-adsorption experiments. In addition, whole-mount immunohistochemistry pre-adsorption experiments were performed to test the specificity of the *Nv*Lgl and *Nv*aPKC antibodies by whole-mount immunohistochemistry. The staining pattern was eliminated in early embryos when pre-incubated antibodies were used (Figure 3B). Unfortunately, the quantity and quality of the peptide for NvPar-1 were not enough to generate the whole-mount controls for this antibody.

### Two phases of Par protein localization during the early development of *N. vectensis*

### N. vectensis *aPKC (*Nv*aPKC) and Par-6 (*Nv*Par-6)*

**Figure 4:**
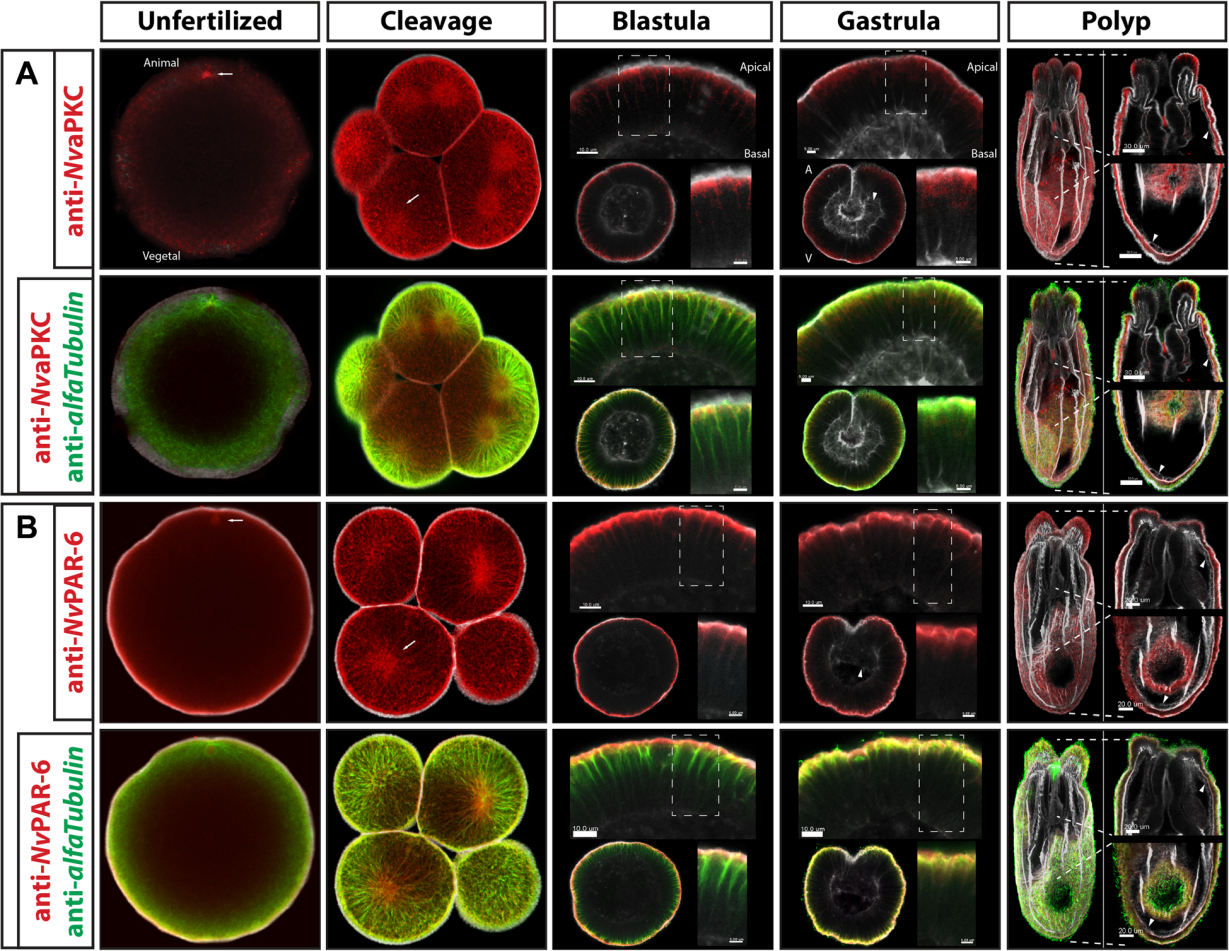
Two phases of *N. vectensis* aPKC (*Nv*aPKC) and Par-6 (*Nv*Par-6) localization. Immunostaining against *Nv*aPKC **(A)** and *Nv*Par-6 **(B)** during different stages of *N. vectensis* development. These proteins do not localize asymmetrically in unfertilized eggs and in the cells of cleavage stages; however, they do localize asymmetrically in the epithelial cells of blastula and gastrula stage embryos. Similar to some bilaterians, *Nv*aPKC and *Nv*Par-6 localize at the apical zone of the cell (insets in blastula and gastrula). Both proteins distribute throughout the microtubule cytoskeleton (labeled with tubulin antibody staining), and labeling was not observed in the endoderm/gastrodermis of gastrula and polyps. White arrows indicate astral/centrosomal and (pro)nuclear structures. White arrowheads indicate the endodermal layer in gastrula and polyp stages. Actin cytoskeleton is shown in gray. Animal/vegetal axis (A/V) and apical/basal axis are indicated. Insets shown in blastula, gastrula, and polyp stages are higher magnification of the zone indicated with the dashed lines. Images ranging from unfertilized to gastrula and insets in polyp stages correspond to a single slice from z-stack series. Image of the whole polyps corresponds to a 3D reconstruction from z-stack series.

In bilaterian animals, aPKC and Par-6 are reported to be localized at the animal pole of the egg and are polarized at the blastomere cortex. The localization of aPKC and Par-6 seems to be conserved among Bilateria. We therefore expected to find similar patterns during the early development of *N. vectensis*. However, immunostaining against *Nv*aPKC and *Nv*Par-6 showed that these proteins are distributed throughout the egg and early embryo with no clear polarization (Figure 4, unfertilized and cleavage). *Nv*aPKC and Par-6 were found to be enriched all around the cortex, in association and with the centrosome and (pro)nuclear structures of the unfertilized and fertilized egg (white arrows in Figure 4, only unfertilized egg is shown). During early cleavage stages, both proteins co-distributed with the mitotic spindle and asters of each cell division and with stable cytoskeletal components (white arrows in Figure 4, cleavage, and Additional file 4). Prior to every mitosis, the localization of these two components was highly concentrated around the nuclear envelope and in cortical membrane domains at locations adjacent to other cells (referred here as regions of cell contacts). During blastula formation, *Nv*Par6 and *Nv*aPKC started to localize at the cortex of the cell and concentrate under the apical membrane, but not the basolateral region. By gastrulation, both proteins localized at the apical zone of the cell, found below the cortical actin (colored as gray in Figure 4). The expression of each protein was only observed in ectodermal tissues with no expression observed in the gastrodermis at all developmental stages investigated up through polyp stages.

**Figure 5:**
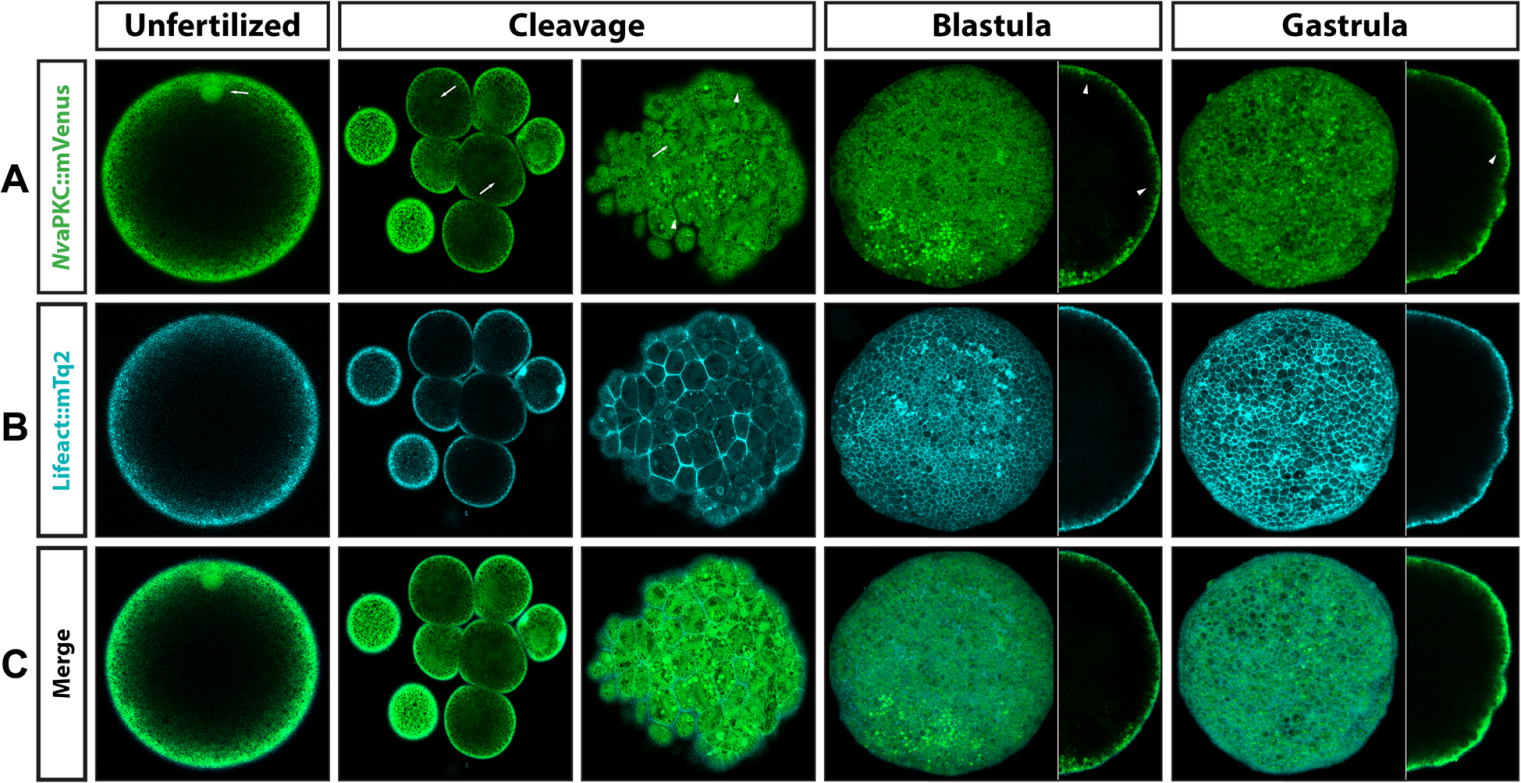
*In vivo* localization of *Nv*aPKC::mVenus during different stages of *N. vectensis* development. The overexpression of *Nv*aPKC::mVenus **(A)** protein, by mRNA microinjection, displays similar patterns observed with the antibody staining against the same protein, and no signal was observed in the endoderm. This messenger was co-injected with Lifeact::mTq2 mRNA **(B)** to visualize the shape of the cells and the apical cortex. As is seen in **(C)**, *Nv*aPKC::mVenus is below the cortical actin during all observed stages. White arrows indicate astral/centrosomal and (pro)nuclear structures. White arrowheads indicate the locations adjacent to other cells (cell contacts) where *Nv*aPKC::mVenus is localized. Images of unfertilized, cleavage, and side panels in gastrula stages correspond to a single slice from z-stack series. Image of the whole blastula and gastrula corresponds to a 3D reconstruction from z-stack series. Side panels in blastula and gastrula stages are a mid section from the z-stack series to show the apical distribution of *Nv*aPKC::mVenus along the A/B axis. See Additional files 5, 6, 7, 8, and 9 for more details of every stage presented here in C.

To confirm our observations obtained by antibody staining, we cloned both *NvaPKC* and *NvPar-6* in frame with the mVenus fluorescent protein. Overexpressed *Nv*aPKC::mVenus showed the same pattern *in vivo* as did the *Nv*aPKC whole-mount antibody stains (Figure 5). No clear polarity was observed in the distribution of this labeled protein during early cleavages (Additional files 5 and 6). *Nv*aPKC::mVenus was distributed throughout the cytoplasm and mitotic apparatus (white arrows in Figure 5A). *Nv*aPKC::mVenus began to localize to regions of cell contacts after several cell cycles (arrowheads in Figure 5A, cleavage, and Additional file 7) and is later displaced to apical zones of the cells. However, its localization in living embryos was diffuse and tended to form small aggregates at the apical cortex of the cell, perhaps due to competition with endogenous unlabeled protein. During blastula (Figure 5A and Additional file 8) and gastrula stages (Figure 5A and Additional file 9), *Nv*aPKC::mVenus localizes to the lateral cell contacts (arrowheads in Figure 5A) and distributes principally to the apical region of the cells (Figure 5A,B,C). Co-injection with Lifeact::mTq2 (Figure 5B), which binds to filamentous actin, revealed that *Nv*aPKC::mVenus (green in Figure 5C) is below the cortical actin (turquoise in Figure 5C) during all stages (Additional files 5, 6, 7, 8, and 9). No endodermal distribution was observed at these stages, supporting the results obtained with antibody staining.

**Figure 6:**
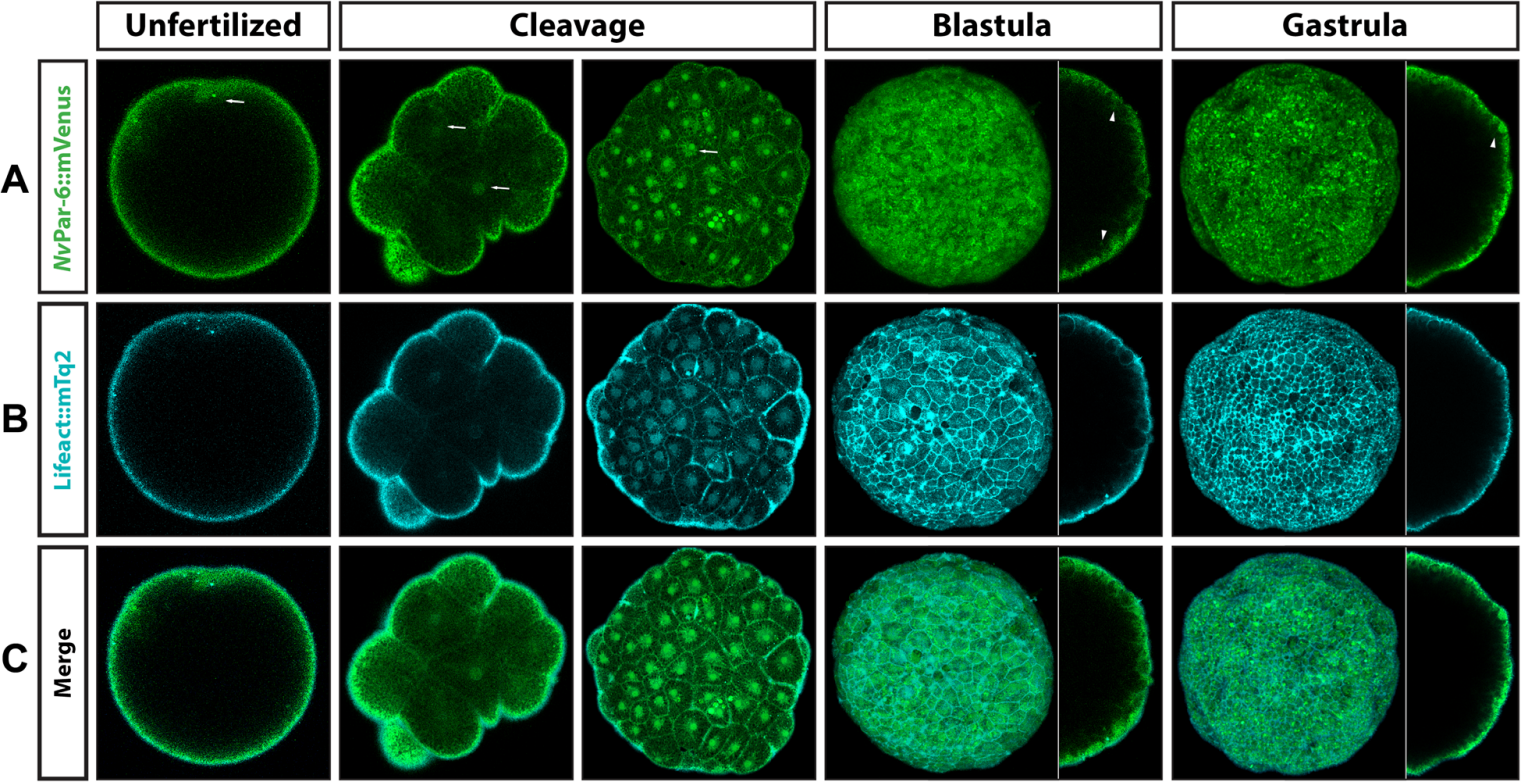
*In vivo* localization of *Nv*Par-6::mVenus during different stages of *N. vectensis* development. The overexpressed *Nv*Par-6::mVenus protein **(A)** displays similar patterns observed with the antibody staining against the same protein, and no signal was observed in the endoderm. Co-injection with Lifeact::mTq2 mRNA **(B)** shows the shape of the cells and their apical cortex. Similar to *Nv*aPKC::mVenus, *Nv*Par-6::mVenus is below the cortical actin during all observed stages **(C)**. White arrows indicate astral/centrosomal and (pro)nuclear structures. White arrowheads indicate the locations adjacent to other cells (cell contacts) where *Nv*Par-6::mVenus is localized. Images of unfertilized, cleavage, and side panels in gastrula stages correspond to a single slice from z-stack series. Image of the whole blastula and gastrula corresponds to a 3D reconstruction from z-stack series. Side panels in blastula and gastrula stages are a mid section from the z-stack series to show the apical distribution of *Nv*Par-6::mVenus along the A/B axis. Additional files 10, 11, 12, 13, and 14 for more details of every stage presented here in C.

The same dynamics seen for *Nv*aPKC::mVenus were observed when we overexpressed *Nv*Par-6::mVenus (Figure 6). No clear polarization or asymmetrical localization was observed during earlier stages (Additional files 10, 11, 12, 13, and 14), while an apical distribution occurred during blastula and gastrula stages (Additional files 13 and 14, respectively). Co-injection with Lifeact::mTq2 suggested that *Nv*Par-6::mVenus does not localize to the cortex of the apical cell membrane. Instead, we observed localization to the cortex of the lateral membrane, at the cell contacts (arrowheads in Figure 6, blastula and gastrula). Further, endodermal distribution was not observed at the studied stages (up through primary polyp formation). Overexpression of these labeled constructs did not appear to have any adverse effects on normal development and injected embryos developed normally up to the primary polyp stage.

### N. vectensis *Lgl (*Nv*Lgl) and Par-1 (*Nv*Par-1)*

**Figure 7:**
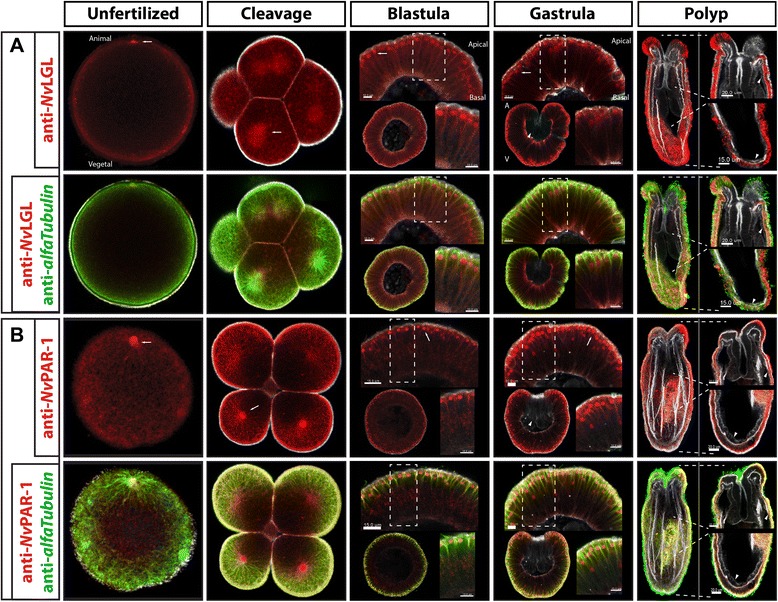
Two phases of *N. vectensis* Lgl (*Nv*Lgl) and Par-1 (*Nv*Par-1) localization. No asymmetric localization of *Nv*Lgl **(A)** and *Nv*Par-1 **(B)** occurs during cleavage stages; however, they localize asymmetrically in the epithelial cells at later stages (insets on blastula and gastrula). Similar to some bilaterians, *Nv*Lgl and *Nv*Par-1 extend throughout basolateral cortex of the cell. Both proteins distribute throughout the microtubule cytoskeleton (labeled with tubulin antibody staining), and no labeling was observed in the endoderm/gastrodermis (gastrula and polyps were observed). White arrows indicate astral/centrosomal and (pro)nuclear structures. White arrowheads indicate the endodermal layer in gastrula and polyp stages. Actin cytoskeleton is shown as gray. Animal/vegetal axis (A/V) and apical/basal axis are indicated. Insets shown in blastula, gastrula, and polyp stages are higher magnification of the zone indicated with the dashed lines. Images ranging from unfertilized to gastrula and insets in polyp stages correspond to a single slice from z-stack series. Image of the whole polyps correspond to a 3D reconstruction from z-stack series.

Lgl and Par-1 are reported to localize to the vegetal cortex of the egg and blastomeres of bilaterians that have been investigated to date [1,2] (Figure 1). Similar to what was observed with *Nv*aPKC and *Nv*Par-6, immunostaining against *Nv*Lgl and *Nv*Par-1 showed that these proteins distribute throughout the egg and early embryo of *N. vectensis* with no clear polarization (Figure 7, unfertilized and cleavage stages). Both proteins were observed around the centrosome and (pro)nuclear structures in unfertilized and fertilized eggs (white arrows in Figure 7A,B, unfertilized). During early cleavage stages, both proteins were co-distributed with the mitotic spindle and asters of each cell division and with stable cytoskeleton components (white arrows in Figure 7, cleavage, and Additional file 4). In addition, both *Nv*Lgl and *Nv*Par-1 were observed along the regions of cell contacts. At later developmental stages, during blastula formation, the cells of the embryo start to display some morphological and molecular aspects of polarization. *Nv*Lgl and *Nv*Par-1 localize to the basolateral cortex, around the nuclei, and in association with mitotic spindles (white arrows in Figure 7, blastula). This pattern is maintained during gastrulation (Figure 7, gastrula) and primary polyp formation (Figure 7, polyp), but the antibody staining of these proteins was observable only in ectodermal tissues (see the ‘Discussion’ section). Strikingly, the staining for the *Nv*Lgl antibody displays an ‘apical’ distribution, similar to *Nv*aPKC. Nocodazole-treated embryos suggest that this ‘apical’ localization of *Nv*Lgl is not stable and it is associated with components of the microtubule cytoskeleton of the mitotic spindle and or nuclei, distinct from *Nv*aPKC localization (Additional file 4).

**Figure 8:**
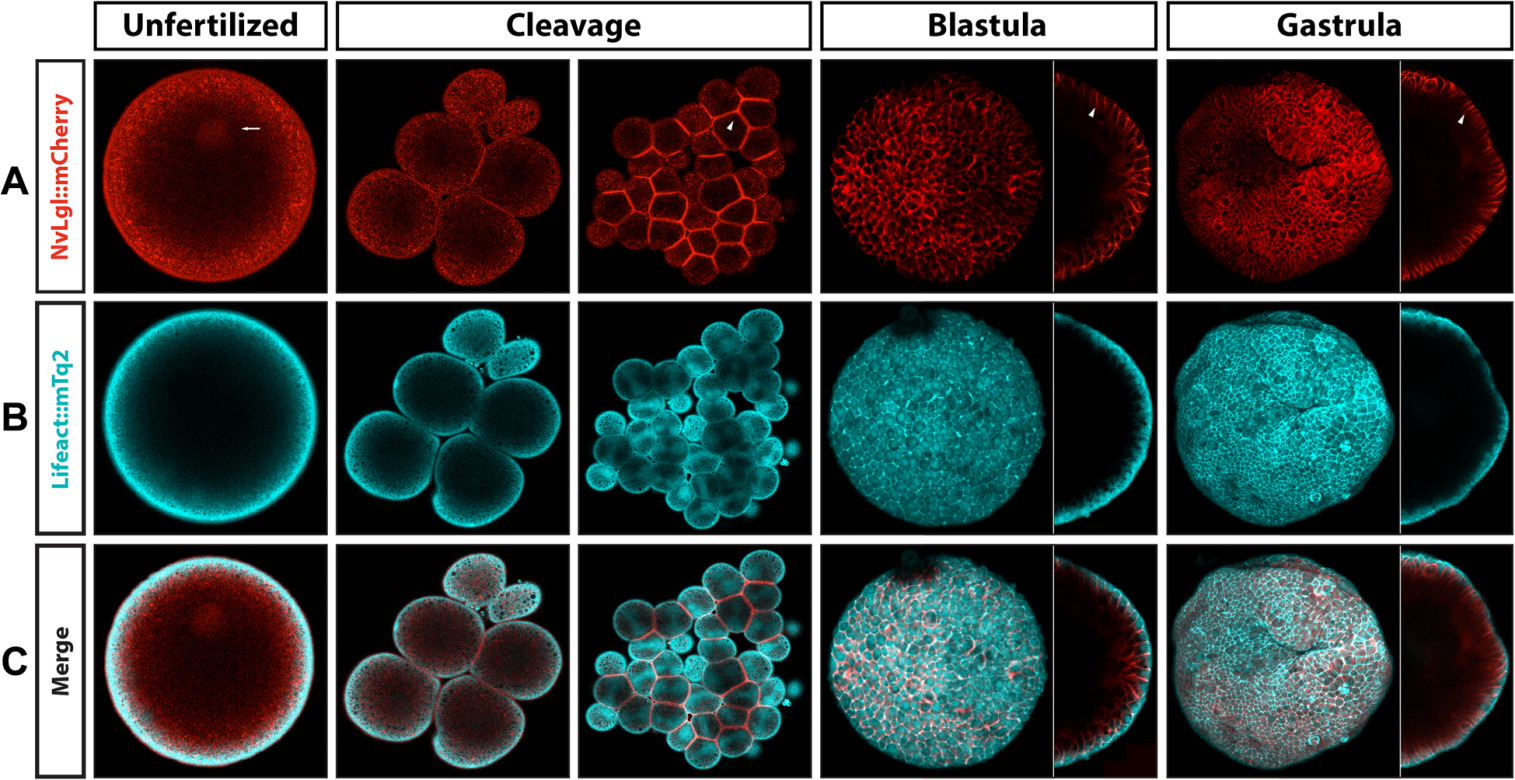
*In vivo* localization of *Nv*Lgl::mCherry during different stages of *N. vectensis* development. The overexpression of *Nv*Lgl::mCherry protein **(A)** displays similar patterns observed with the antibody staining against the same protein, and no signal was observed in the endoderm. Lifeact::mTq2 mRNA **(B)** was co-injected to visualize the shape of the cells and the apical cortex. In **(C)**, *Nv*Lgl::mCherry is always expressed below the cortical actin and localized in the lateral cortex during all observed stages. White arrows indicate astral/centrosomal and (pro)nuclear structures. White arrowheads indicate the locations adjacent to other cells (cell contacts) where *Nv*Lgl::mCherry is localized. Images of unfertilized, cleavage, and side panels in gastrula stages correspond to a single slice from z-stack series. Image of the whole blastula and gastrula corresponds to a 3D reconstruction from z-stack series. Side panels in blastula and gastrula stages are a mid section from the z-stack series to show the basolateral distribution of *Nv*Lgl::mCherry along the A/B axis. See Additional files 15, 16, 17, 18, and 19 for more details of every stage presented here in C.

Similar results were observed upon overexpression of *Nv*Lgl::mCherry (Figure 8), which was displaced to basolateral zones when the embryo formed epithelial tissues (Figure 8A,B,C). At earlier stages, as we observed with *Nv*Lgl antibody staining, *Nv*Lgl::mCherry was not localized cortically and was distributed throughout the cytoplasm and mitotic apparatus of the cells (white arrow in Figure 8A and Additional files 15 and 16). After several cleavages, *Nv*Lgl::mCherry began to localize to the regions of cell contacts but no clear polarization was observed (arrowhead in Figure 8A and Additional file 17). However, when the embryo was raised to the blastula stage and was became a monolayer of epithelial cells, *Nv*Lgl::mCherry was displaced to the basolateral zone of the cells (Additional file 18) where it remained through gastrulation stages (Figure 8, gastrula, and Additional file 19). Co-injection with Lifeact::mTq2 showed the absence of *Nv*Lgl::mCherry in apical domains (Figure 8C and Additional files 15, 16, 17, 18, and 19). Concurrent with results from other Par system components, endodermal expression of *Nv*Lgl::mCherry was not observed at the stages under investigation. Confirming results that were obtained by previously described immunohistochemical experiments, *Nv*Lgl::mCherry injected embryos (Additional file 20) showed the co-distribution of anti-*Nv*Lgl and anti-*Nv*Par-1 with anti-mCherry antibody.

### Polarization of *N. vectensis* Par-3 (*Nv*Par-3) *in vivo*

**Figure 9:**
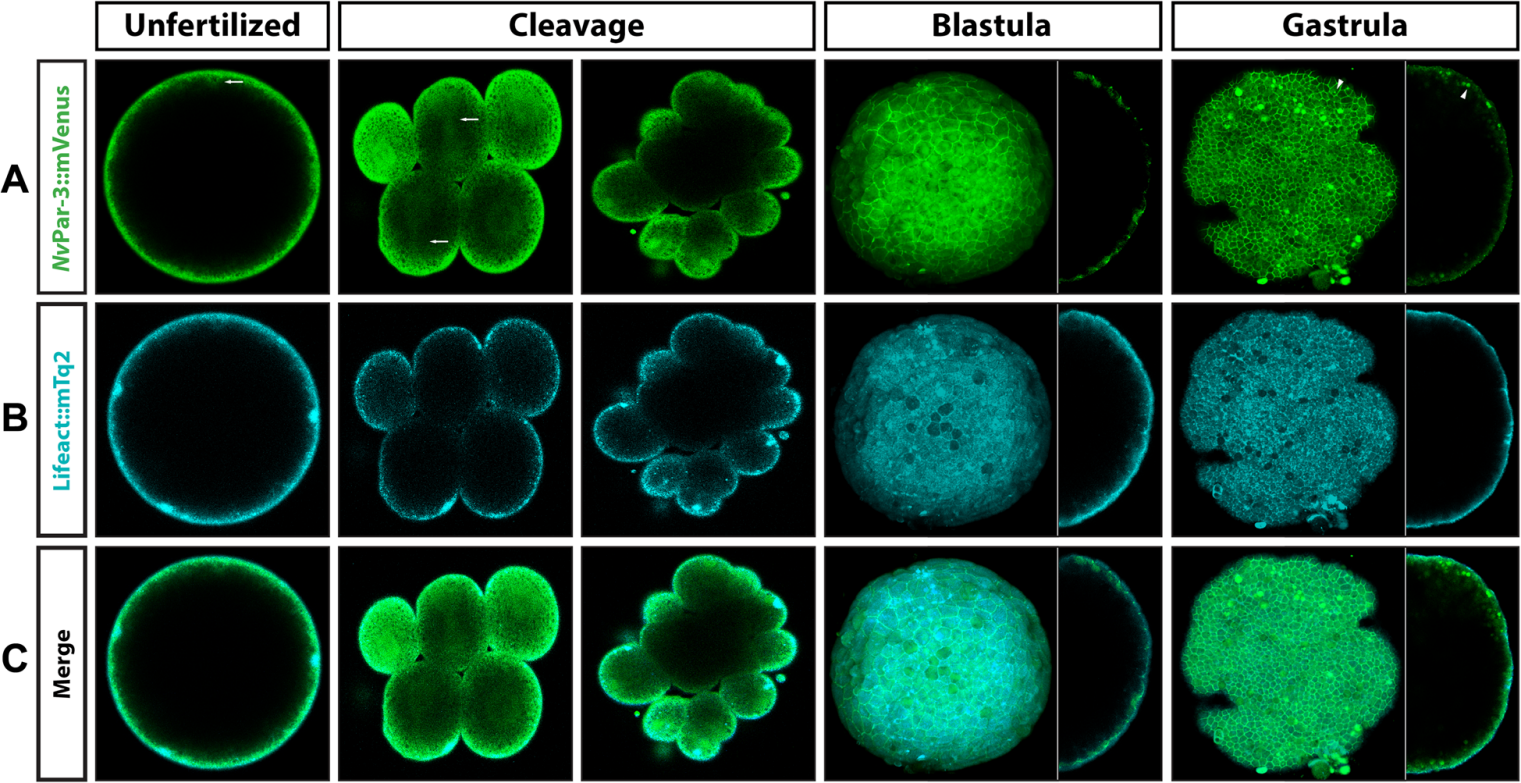
*In vivo* localization of *Nv*Par-3::mVenus during different stages of *N. vectensis* development. The overexpression of *Nv*Par3::mVenus **(A)** protein, by mRNA microinjection, displays two phases of distribution, similar to what was observed in *Nv*aPKC and *Nv*Par-6: no asymmetric localization during earlier cleavage stages but differential localization along the A/B axis of the cells during later stages (blastula and gastrula). Interestingly, *Nv*Par3::mVenus localizes only in the apico-lateral zone of the cells, presumably at the cell-cell junctions complex and centrosomes (arrowhead), and no signal was observed in the endoderm. *Nv*Par3::mVenus was co-injected with Lifeact::mTq2 mRNA **(B)** to visualize the shape of the cells and the apical cortex. As is seen in **(C)**, *Nv*Par3::mVenus is below the cortical actin during all observed stages. White arrows indicate astral/centrosomal and (pro)nuclear structures. Images of unfertilized, cleavage, and side panels in gastrula stages correspond to a single slice from z-stack series. Image of the whole blastula and gastrula corresponds to a 3D reconstruction from z-stack series. Side panels in blastula and gastrula stages are a mid section from the z-stack series to show the apico-lateral distribution of *Nv*Par3::mVenus along the A/B axis. See Additional files 21, 22, 23, 24, and 25 for more details of every stage presented here in C.

Although we do not have specific antibodies against *Nv*Par-3, we were able to follow this protein *in vivo* by overexpressing mVenus-tagged mRNA (Figure 9). The use of reporter gene constructs with other Par system components produced results matching those obtained through immunohistochemistry. We are therefore confident that this fluorescent reporter approach would accurately reflect the endogenous protein distribution of *Nv*Par-3. Similar to observed results for *Nv*aPKC::mVenus and *Nv*Par-6::mVenus localization, *Nv*Par-3::mVenus did not display any polarization at very early stages: it distributed throughout the cytoplasm and mitotic apparatus of the cells in unfertilized eggs and cleavage stages (white arrows in Figure 9A and Additional files 21 and 22). After a few cleavages, however, up through gastrulation, *Nv*Par-3::mVenus became localized only at the apico-lateral cortex, below actin localization at the region of cell contacts (Figure 9C and Additional file 23). This result suggests a possible association between *Nv*Par-3 and junctional complexes, which may limit the distribution of Par complexes. This pattern became more obvious in epithelial cells as the embryo reached the blastula and gastrula stages (Figure 9A,B,C and Additional files 24 and 25). Interestingly, *Nv*Par-3::mVenus also appears to become localized to areas surrounding the centrosomes (arrowhead in Figure 9 and Additional file 26), as has been described for some bilaterians [21,58]. Ectopic expression of *Nv*Par-3::mVenus did not appear to have any detrimental role on development as all treated embryos developed in a normal fashion to the primary polyp stage. The reported expression pattern of *Nv*Par-3::mVenus was observed in all injected embryos.

### Defining apical and basolateral limits using multiple fluorescent protein reporters

**Figure 10:**
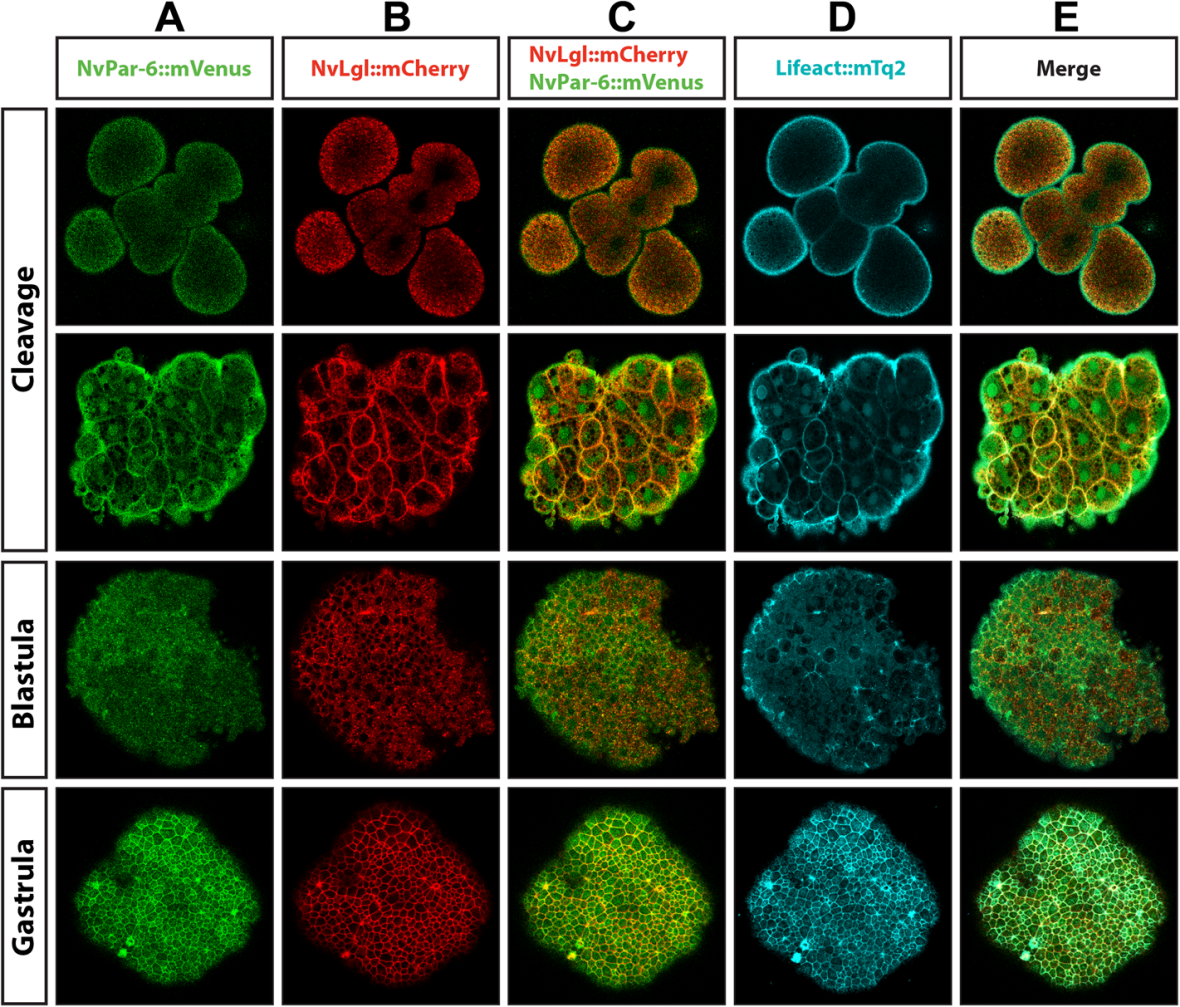
*In vivo* embryonic localization of *Nv*Par-6::mVenus and *Nv*Lgl::mCherry during different stages of *N. vectensis* development. Co-injection of *Nv*Par-6::mVenus **(A)** and *Nv*Lgl::mCherry **(B)** confirms our observations for the localization of both proteins through separate experimental approaches. Throughout *N. vectensis* development, both proteins separate into distinct domains **(C)**. Lifeact::mTq2 mRNA **(D)** was used to visualize the shape of the cells and the apical cortex. *Nv*Par-6::mVenus and *Nv*Lgl::mCherry were always observed below the cortical actin during all observed stages **(E)**. Images of cleavage and blastula stages correspond to a single slice from z-stack series. Images of the gastrula stage correspond to a 3D reconstruction from z-stack series. See Additional files 27, 28, 29, and 30 and Figure 11 for more details of every stage presented here.

**Figure 11:**
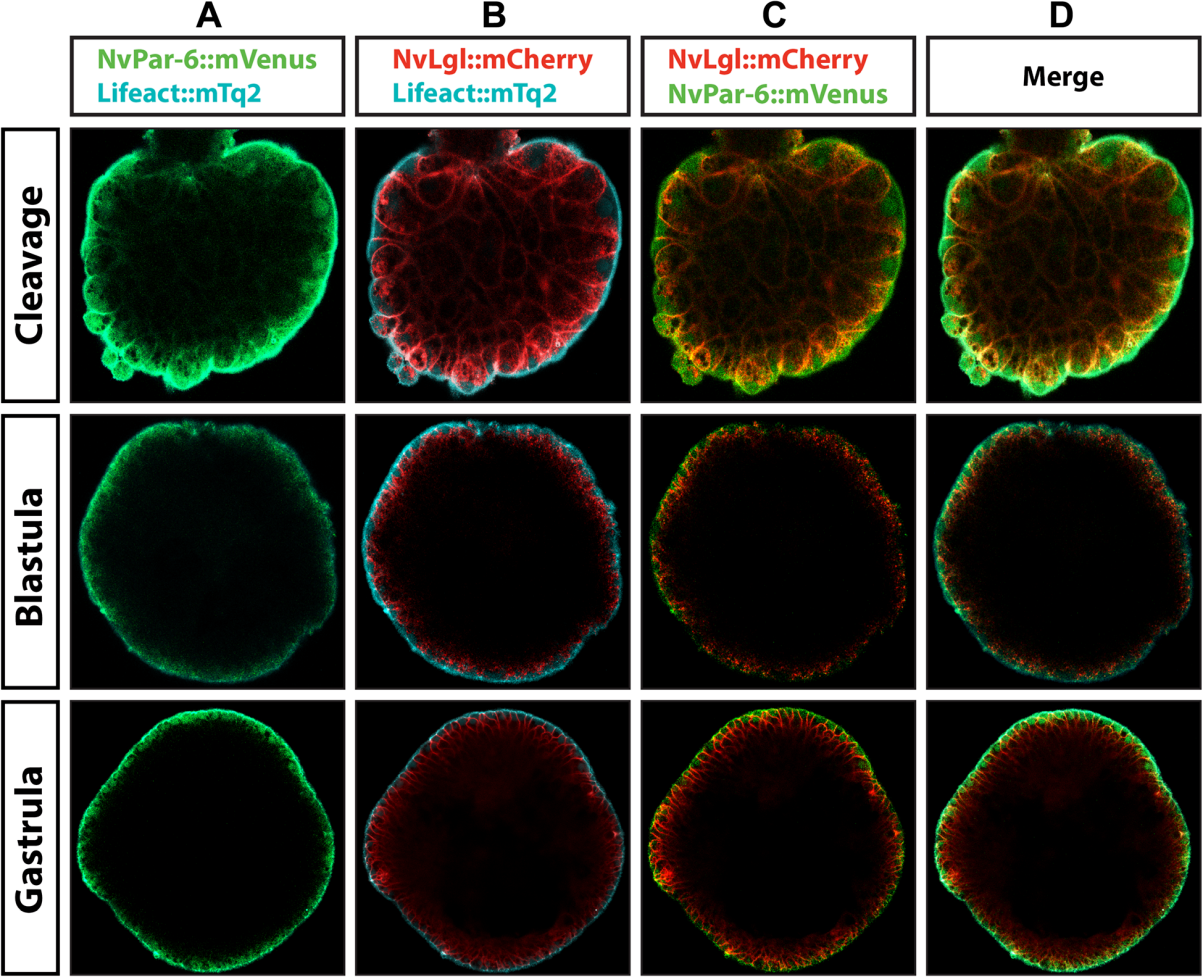
*In vivo* cortical localization of *Nv*Par-6::mVenus and *Nv*Lgl::mCherry during different stages of *N. vectensis* development. z-stack sections from the stages are shown in Figure 10. Beginning at later cleavage stages, both *Nv*Par-6::mVenus **(A)** and *Nv*Lgl::mCherry **(B)** began to distribute in different regions inside the cells **(C)** below the cortical actin labeled with Lifeact::mTq2 **(D)**: *Nv*Par-6::mVenus was always observed towards the apical side while *Nv*Lgl::mCherry moved basolaterally in non-overlapping domains.

With the intention to verify our observations of immunostaining and overexpression of the individual Par system proteins, we simultaneously overexpressed reporter gene constructs for three different labeled proteins. Figures 10 and 11 show the results obtained after co-injecting fluorescent reporter mRNA for the actin-binding protein Lifeact::mTq2, *Nv*Lgl::mCherry and *Nv*Par6::mVenus, imaged at different stages of development. At early cleavage stages (Figures 10 and 11), *Nv*Par6::mVenus (Figure 10A) and *Nv*Lgl::mCherry (Figure 10B) co-distributed throughout the cytoplasm of the cells (Figure 10C), with cell boundaries demarcated by Lifeact::mTq2 (Figure 10D and Additional file 27). As development proceeded, *Nv*Par6::mVenus and *Nv*Lgl::mCherry became localized to the lateral membrane, where cells contact each other, and subsequently began to migrate towards different poles of the cell (Additional file 28 and cleavage in Figure 11C). Then, when the embryo reached blastula and gastrula, the epithelial tissue was forming, the cells became highly polarized, and both Par system proteins localized to opposite domains: *Nv*Par6::mVenus (Figure 11A) always localized towards the apical side while *Nv*Lgl::mCherry (Figure 11B) moved basolaterally in non-overlapping domains (Figure 11C, Additional files 29 and 30). Interestingly, at early developmental stages, the fluorescent signals for both Par system proteins were observed in close proximity to one another, suggesting a possible association between these two proteins (data not shown). Similar results were observed by co-immunostaining with *Nv*Lgl::mCherry and anti-*Nv*Par-6 (Additional file 20).

**Figure 12:**
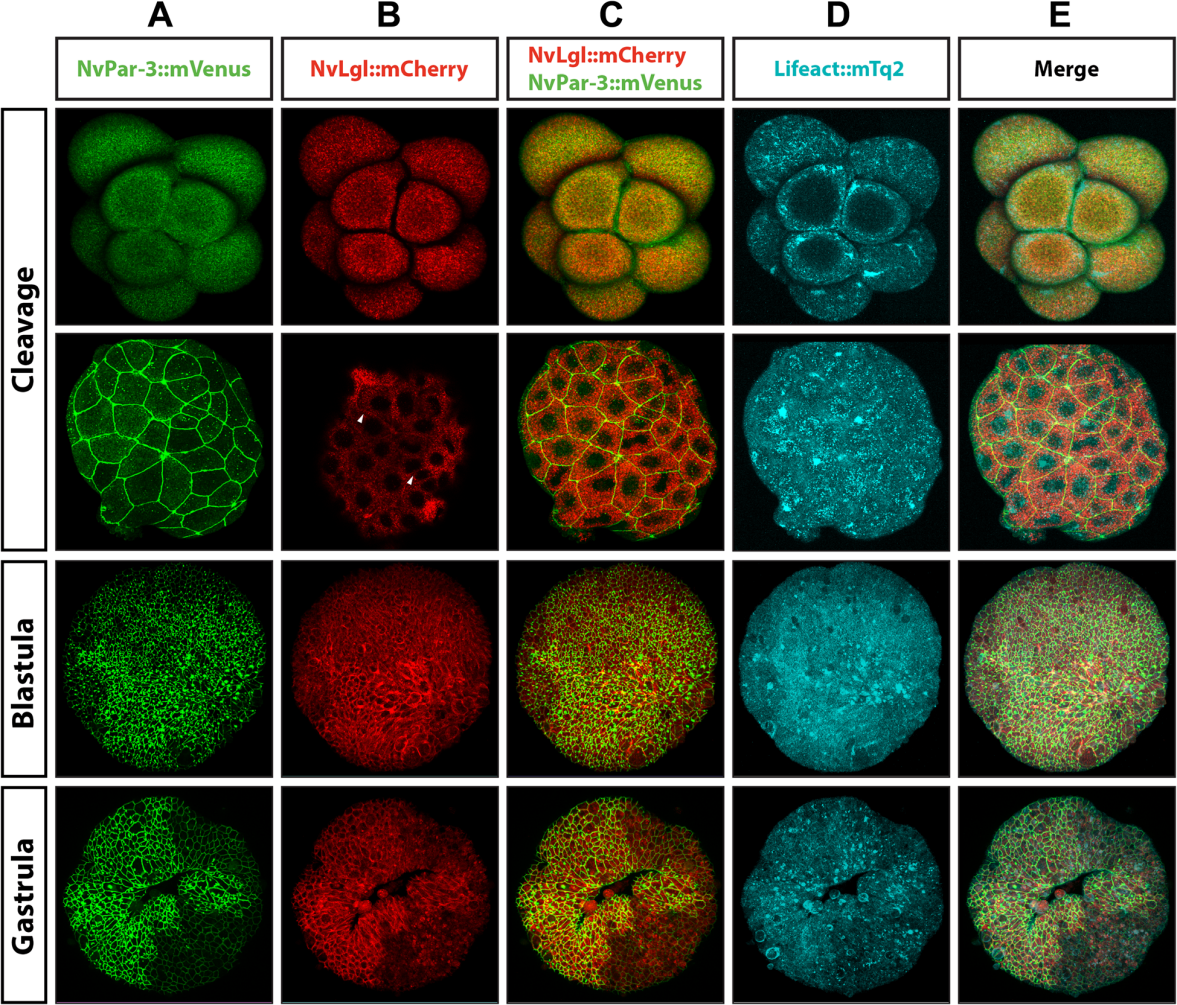
*In vivo* embryonic localization of *Nv*Par-3::mVenus and *Nv*Lgl::mCherry during different stages of *N. vectensis* development. Co-injection of *Nv*Par-3::mVenus **(A)** and *Nv*Lgl::mCherry **(B)** displays the same distribution observed when we overexpressed each protein separately: co-distribution during earlier stages but asymmetric localization in the cells of later stages **(C)**. Lifeact::mTq2 mRNA **(D)** was used to visualize the shape of the cells and the apical cortex. *Nv*Par-3::mVenus and *Nv*Lgl::mCherry were observed below the cortical actin during all observed stages **(E)**. White arrowheads indicate the localization of *Nv*Lgl::mCherry at the cell contacts. All images correspond to a 3D reconstruction from z-stack series. See Additional files 31, 32, 33, and 34 and Figure 13 for more details of every stage presented here.

**Figure 13:**
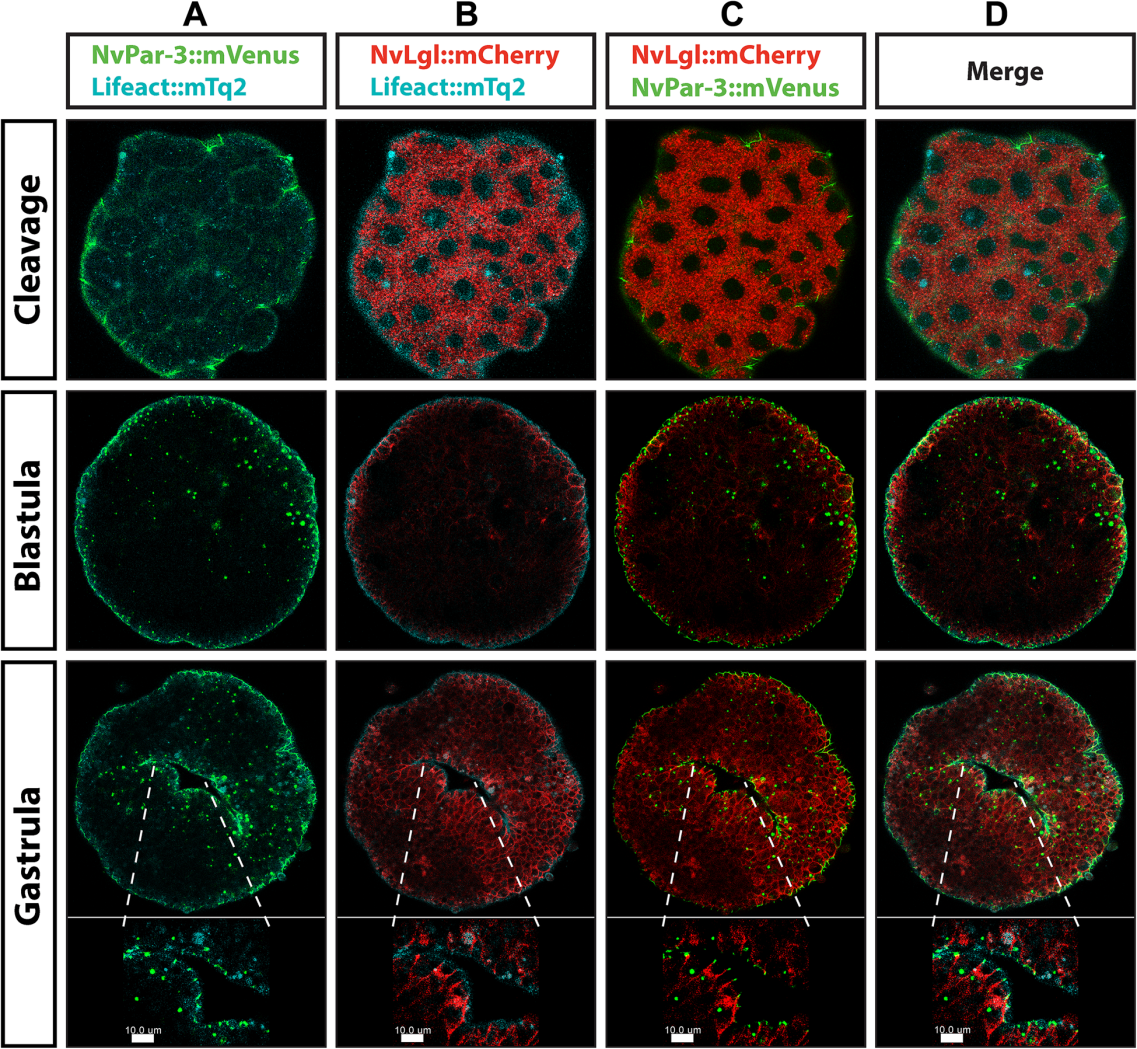
*In vivo* cortical localization of *Nv*Par-3::mVenus and *Nv*Lgl::mCherry during different stages of *N. vectensis* development. z-stack sections from the stages are shown in Figure 12. *Nv*Par-3::mVenus **(A)** and *Nv*Lgl::mCherry **(B)** distributed in different regions inside the cells **(C)** below the cortical actin labeled with Lifeact::mTq2 **(D)**: *Nv*Par-3::mVenus was always observed towards the apico-lateral membrane while *Nv*Lgl::mCherry moved basolaterally. *Nv*Par-3::mVenus is localized at the cell-cell contacts where the separation between apical and basolateral regions is clearly defined (C, gastrula insets). Insets show the asymmetric distribution of *Nv*Par-3::mVenus and *Nv*Lgl::mCherry in the cells of ectodermal epithelium at the gastrula stage. The magnified region was selected to represent the asymmetric distribution of those proteins and is not intended for function implication.

Using *Nv*Par3::mVenus as an apical marker, we simultaneously co-injected mRNAs for *Nv*Par3::mVenus, *Nv*Lgl::mCherry, and Lifeact::mTq2 (Figures 12 and 13), in order to see how both Par system proteins, *Nv*Par3::mVenus (Figures 12A and 13A) and *Nv*Lgl::mCherry (Figures 12B and 13B), localized when they were expressed together (C in Figures 12 and 13). As was expected from the results previously shown, both proteins displayed a cytoplasmic distribution during earlier cleavage stages (Additional file 31). After a few cell cycles (Additional file 32), *Nv*Lgl::mCherry became localized to the site of cell contacts (arrowheads in Figure 12B), similar to the localization of *Nv*Par3::mVenus (Figure 12A). During later development (Additional file 33), the localization of *Nv*Lgl::mCherry extended along the basolateral cortex (blastula and gastrula stages in Figure 13B). *Nv*Par3::mVenus was always observed at the apical cell cortex and did not overlap with *Nv*Lgl::mCherry but was always absent from the most apical membrane labeled by Lifeact::mTq2 (see insets of gastrula stage in Figure 13 and Additional file 34).

## Discussion

There has been great interest in the development and evolution of cell polarity in metazoan lineages. To date, these processes have been thoroughly described in the Bilateria, the clade that includes all the best studied animal model systems. During bilaterian development, the same set of protein interactions, described here as the Par system, is utilized to organize the polarity of both the embryonic cells and epithelial cells. As would be expected, these proteins are also present in Cnidaria [44,59], the sister group of Bilateria, and their role in establishing cell polarity is investigated here for the first time.

### Comparison of the Par system between Cnidaria and Bilateria

Polarization of the oocyte during oogenesis is a critical step for the proper embryonic development of many animals. Cnidarian eggs and embryos have been shown to be highly polarized; the animal pole always gives rise to the site of gastrulation and gives rise to the oral pole [32,47]. This polarization is generated during oogenesis [60] where several maternal determinants such as organelles, mRNAs, and proteins are localized to the animal pole. For example, Dishevelled, Flamingo, and Strabismus (components of the Wnt signaling pathway) are asymmetrically localized to the animal pole prior to the first cleavage of *N. vectensis* egg [31,32,61], and these proteins also serve to polarize the early embryo, have essential roles specifying the site of gastrulation [31,32], and polarize epithelial cells [31]. A similar situation has been described in the vegetal pole of bilaterian embryos, suggesting that bilaterian and cnidarian animals share conserved mechanisms to polarize their cells. Thus, we expected to find evidence that the Par system would also play a role in establishing the polarity of *N. vectensis* embryos as they do in bilaterian embryos. However, this does not appear to be the case.

**Figure 14:**
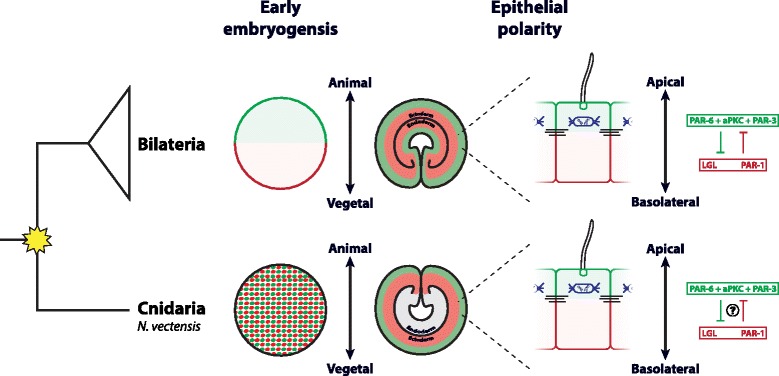
Par system co-opted into early embryonic stages at the base of the Bilateria. The Par system described for bilaterian animals is deployed during early stages to establish cell polarity, maintained during development, and later used to polarize the cells of epithelial tissues. A different scenario was observed for the embryonic and tissue cell polarity in Cnidaria: Par system proteins are not asymmetrically distributed during early development of *N. vectensis*, but they later become polarized in the cells of the epithelia. This suggests that the Par system, a critical regulator of tissue cell polarity, was co-opted into establishing early embryonic polarity at the base of the Bilateria (yellow star).

All Par system components of *N. vectensis* examined here show distinct associations with the microtubule cytoskeleton but no asymmetry in their distribution relative to embryonic axis formation in the cells of early embryo. Unlike embryonic cell polarity, components of the Par system appear to serve a similar role in establishing the polarity of epithelial cells in Cnidaria and Bilateria. Both clades develop epithelia consisting of a sheet of polarized cells that rest on a basal lamina and are joined by belt-like junctions, which define apical and basolateral membrane domains. During the development of the blastula stage, proteins of the *N. vectensis* Par system gradually localize in different spatial domains of the cells. During these stages, the cells of the embryo organize themselves into a single-layered epithelium that brings cells into closer proximity with one another [47,62]. Within this epithelization process, all cells of the ectodermal epithelia, at the blastula and gastrula stages, become polarized, and Par proteins adopt an asymmetric distribution (Figures 4, 5, 6, 7, 8, 9, 10, 11, 12, and 13). Similar to what has been reported in some bilaterians, *Nv*Lgl and *Nv*Par-1 localize in the basolateral cortex while *Nv*aPKC, *Nv*Par-6, and *Nv*Par-3 localize to the apical zone of the cell (Figure 14). Two separate experimental approaches were used to visualize the localization of these Par system components, each resulting in similar observations of their spatial distribution. Results obtained both immunohistochemically and in living embryos using fluorescently tagged reporter proteins, demonstrated that our observations are all consistent with the ability of our four antibodies to detect their target proteins. Three of the antibody signals (*Nv*aPKC, *Nv*Lgl, and *Nv*Par-1) where inhibited by competition assays with their injected antigen, and the fourth confirmed by the co-immunostainig with *Nv*Lgl::mCherry, see Additional file 20). It should be noted that the expression of all fluorescently tagged PAR components by mRNA injection had not affect on normal development.

The ectodermal epithelium formed during embryogenesis in *N. vectensis* is composed of columnar cells, which are joined together by a belt of adherens junctions [38,47,62]. In contrast, following gastrulation, endodermal cells lose their columnar shape and become disorganized, resulting in fewer (or none) and shorter adherens junctions, leaving more spaces between cells [62]. Curiously, our results, either by immunohistochemistry or using living embryos, indicate that no components of the Par system are expressed in endodermal tissue, even at later stages (Figures 4 and 7). Since Par proteins are expressed throughout the blastula, it might be possible that loss of cell-cell adhesion in endodermal cells induces the degradation of Par proteins. During the formation of bilaterian epithelia, the Par system is essential for the formation and maintenance of cell-cell junctions (tight junctions in vertebrates and adherent junctions in invertebrates) [63-67]. In addition, the adhesions between cells become necessary to maintain A/B polarity and Par protein asymmetry [5,16,68]. Therefore, the disruptions of junctional complexes may lead the disruption of the A/B polarity and Par proteins asymmetry. Similar interdependency has been described in different bilaterian animals [10,16], suggesting the conservation of some mechanisms involved in epithelia formation. The absence of Par protein expression has been reported previously in the immature endoderm of fish [68], and in mesodermally derived MDCK cells, where Snail expression disrupts the localization of the Par complexes and A/B polarity by repression of Crumbs3 transcription [69]. Interestingly, gastrodermal cells of *N. vectensis* exhibit molecular profiles that are similar to, and arguably synonymous with, both bilaterian endoderm and mesoderm (including Snail of [62]). It is therefore possible that the absence (or degradation) of Par system components in the gastrodermis of *N. vectensis* is a reflection of its dual functional specification. While theoretically interesting, this point, and its associated biological implications, must be vetted by extensive functional analysis.

### The evolution of cell polarity and the Par system

Our findings indicate that the bilaterian Par system shares a role in polarizing epithelial cells in cnidarians, but not in the cells of early embryo. If the situation in *N. vectensis* is representative of other prebilaterians, it would suggest that the polarization of early embryonic cells by the Par system could have arisen at the base of the Bilateria. The most parsimonious evolutionary explanation for this pattern is that the Par system played a role in establishing the epithelial cell polarity in the most recent common ancestor of Cnidaria and Bilateria and that the Par system was then co-opted into an early role in bilaterian embryogenesis.

The asymmetric localization of the proteins involved in the Par system is a developmental process that seems to be conserved (Figure 1); however, there is some evidence in other bilaterian systems that these genes are integrated into the embryonic system at different developmental stages. In *Caenorhabditis elegans*, the first cue of asymmetry arises after fertilization triggered by cytoskeleton reorganization, induced by the sperm centrosome [23]. Similar events have been described for the leech *Helobdella robusta*, with the exception that the asymmetrical localization of Par proteins is observable at two-cell stage [22]. On the other hand, in *Drosophila*, asymmetric localization of Par proteins takes place during oogenesis and is induced by some signals from the follicle cells that surround the vegetal pole [3,29]. During mouse embryogenesis, the first clues of polarity are observed during oogenesis, when Par-6 localizes at the animal pole of the oocyte [11,27,70]. In *Xenopus*, membrane polarization occurs during oogenesis and an asymmetric localization of Par system components is observed when the oocyte is completely mature [4,10,71]. In a different way, sea urchin embryos show asymmetry of Par proteins along the A/V axis at the 16-cell stage [72]; however, aPKC and Par6 are already polarized in blastomeres at the two-cell stage [28]. In all of these bilaterian organisms*,* the Par system appears to be causally involved in establishing cellular polarity, and functional disruption of the Par system components gives rise to similar phenotype when inactive in early bilaterian embryos: when disrupted, Par proteins distribute throughout the embryo without clear polarization and associate with cortical and cytoplasmic components such as centrosomes and nuclei [16]. Similar distributions were observed in equivalent stages for all proteins studied in this work (*Nv*aPKC, *Nv*Par-3, *Nv*Par-6, *Nv*Par-1, and *Nv*Lgl) during early embryogenesis of *N. vectensis* (see Figures 4, 5, 6, 7, 8, 9, 10, 11, 12, and 13; cleavage stages). Furthermore, the overexpression of any of the Par system components by mRNA injection did not disrupt the formation of polarized epithelia, and the overexpressed protein displayed the same distribution observed with the antibody staining. This suggests that Par system might be inactive in *N. vectensis* blastomeres.

One thing of interest is that when par complex proteins are mutated/knocked down in bilaterians, it affects the organization of the cleavage program and the segregation of maternal components [28,29,45,46]. Most cnidarians, including *N. vectensis*, have a ‘chaotic’ cleavage program in which cells divide asynchronously and asymmetrically in a unique way from embryo to embryo. Our results raise the possibility that this chaotic development observed in *N. vectensis* embryogenesis is correlated to the symmetric distribution of Par proteins and that they were not related to the processes regulating early cleavages. Perhaps the co-option of Par complex proteins facilitated the more stereotyped cleavage patterns seen in many bilaterian forms.

## Conclusions

Early embryogenesis in many bilaterians is characterized by a stereotypical pattern of cell divisions directed by the association of different molecular pathways (Figure 1A). These interactions act to polarize the egg and embryonic cells and are used later to polarize the cells of epithelial tissues (Figure 14). Here, we report an initial characterization of the spatial and temporal dynamics of the Par system proteins in a non-bilaterian metazoan, the cnidarian *N. vectensis*. Similar to the situation seen in bilaterian animals, proteins of the Par system are localized asymmetrically in the ectodermal epithelium of *N. vectensis*, but asymmetric localization was not observed during cleavage stages (Figure 14). These results suggest that different mechanisms establish cellular polarity in *N. vectensis* embryos and demonstrate that although these proteins are used to organize epithelial cell polarity later in development, they are not utilized to organize early embryonic cells in the same way they do in later evolving animals. We hypothesize that the ancestral Par system could have functioned to maintain cell-cell contacts and generate polarized epithelia. Further, it is likely that currently unidentified components, which are necessary to direct Par system polarization (Figure 1A), are not present or non-functional during early embryogenesis of *N. vectensis*. Collectively, this data suggests that the molecular regulators of cell polarity in tissues were co-opted into early embryonic cells at the base of the Bilateria (Figure 14). In this scenario, an activated Par system could have shifted to earlier stages directing the polarization, plane of cell division, and the partitioning of the maternal components in the cytoplasm. Interestingly, the same polarity system that operates in the epidermis of *N. vectensis* does not seem to be involved with gastrodermal polarity. Future work will be directed to assess the evolutionary significance of this, and the function of Par system and its interaction with other polarity pathways during the early development of *N. vectensis*.

## Additional files

Additional file 1:
**Embryos of** ***N. vectensis*** **do not have distinguishable cell boundaries during cleavage stages.** Immunostaining against alpha-tubulin labels more than one mitotic apparatus (arrowheads) enclosed by the same cell membrane. Additional file 2:
**Random distribution patterns of maternal mRNA at four-cell stages.** Maternal mRNA of *Nv*aPKC, *Nv*Par-3, *Nv*Par-6, *Nv*Par-1, and *Nv*Lgl are randomly distributed during the four-cell stage of *N. vectensis* embryo (their pattern expression is not consistent). This suggests that mRNA asymmetric distribution in *N. vectensis* is not correlated with the A/V polarity. Additional file 3:
**Specificity of** ***N. vectensis*** **polyclonal affinity-purified antibodies by western blotting.** Western blots of *N. vectensis* gastrula extracts using specific antibodies against *Nv*aPKC (A), *Nv*Lgl (B), *Nv*Par-6 (C), and *Nv*Par-1 (D). A red arrow indicates the expected band of each protein. Additional file 4:
**The protein localization of** ***Nv*** **aPKC and** ***Nv*** **Lgl is associated with stable components of microtubule and actin cytoskeleton.** Protein localization of *Nv*aPKC and *Nv*Lgl is associated with stable components of microtubule and actin cytoskeleton. We tested the cytoskeletal dependency of intracellular localization by treating during 1-h embryos with drugs against the polymerization of microtubules (nocodazole 10 μM/1 h) and actin cytoskeleton (cytochalasinB 5 μM/1 h). During earlier stages, the localization of both *Nv*Lgl and *Nv*aPKC is not affected by either treatment, with no clear polarization observed. Likewise, during gastrulation, the basolateral localization of *Nv*Lgl and the apical distribution of *Nv*aPKC remain, suggesting that the localization of both proteins is associated with either stable microtubules or actin cytoskeleton. Colors green and gray are used to show tubulin and actin labeling, respectively. Additional file 5:
***In vivo*** **localization of** ***Nv*** **aPKC::mVenus in unfertilized egg of** ***N. vectensis*** **development.** z-stack series corresponding to an unfertilized egg co-expressing *Nv*aPKC::mVenus and Lifeact::mTq2 proteins (shown in Figure 5C, unfertilized). Additional file 6:
***In vivo*** **localization of** ***Nv*** **aPKC::mVenus in early cleavage embryo of** ***N. vectensis*** **development.** z-stack series corresponding to an early embryo co-expressing *Nv*aPKC::mVenus and Lifeact::mTq2 proteins (shown in Figure 5C, cleavage). Additional file 7:
***In vivo*** **localization of** ***Nv*** **aPKC::mVenus in late cleavage embryo of** ***N. vectensis*** **development.** z-stack series corresponding to a late embryo co-expressing *Nv*aPKC::mVenus and Lifeact::mTq2 proteins (shown in Figure 5C, cleavage). Additional file 8:
***In vivo*** **localization of** ***Nv*** **aPKC::mVenus in blastula stage of** ***N. vectensis*** **development.** z-stack series corresponding to a blastula stage embryo co-expressing *Nv*aPKC::mVenus and Lifeact::mTq2 proteins (shown in Figure 5C, blastula). Additional file 9:
***In vivo*** **localization of** ***Nv*** **aPKC::mVenus in gastrula stage of** ***N. vectensis*** **development.** z-stack series corresponding to a gastrula stage embryo co-expressing *Nv*aPKC::mVenus and Lifeact::mTq2 proteins (shown in Figure 5C, gastrula). Additional file 10:
***In vivo*** **localization of** ***Nv*** **Par-6::mVenus in unfertilized egg of** ***N. vectensis*** **development.** z-stack series corresponding to an unfertilized egg co-expressing *Nv*Par-6::mVenus and Lifeact::mTq2 proteins (shown in Figure 6C, unfertilized). Additional file 11:
***In vivo*** **localization of** ***Nv*** **Par-6::mVenus in early cleavage embryo of** ***N. vectensis*** **development.** z-stack series corresponding to an early embryo co-expressing *Nv*Par-6::mVenus and Lifeact::mTq2 proteins (shown in Figure 6C, cleavage). Additional file 12:
***In vivo*** **localization of** ***Nv*** **Par-6::mVenus in late cleavage embryo of** ***N. vectensis*** **development.** z-stack series corresponding to a late embryo co-expressing *Nv*Par-6::mVenus and Lifeact::mTq2 proteins (shown in Figure 6C, cleavage). Additional file 13:
***In vivo*** **localization of** ***Nv*** **Par-6::mVenus in blastula stage of** ***N. vectensis*** **development.** z-stack series corresponding to a blastula stage embryo co-expressing *Nv*Par-6::mVenus and Lifeact::mTq2 proteins (shown in Figure 6C, blastula). Additional file 14:
***In vivo*** **localization of** ***Nv*** **Par-6::mVenus in gastrula stage of** ***N. vectensis*** **development.** z-stack series corresponding to a gastrula stage embryo co-expressing *Nv*Par-6::mVenus and Lifeact::mTq2 proteins (shown in Figure 6C, gastrula). Additional file 15:
***In vivo*** **localization of** ***Nv*** **Lgl::mCherry in unfertilized egg of** ***N. vectensis*** **development.** z-stack series corresponding to an unfertilized egg co-expressing *Nv*Lgl::mCherry and Lifeact::mTq2 proteins (shown in Figure 8C, unfertilized). Additional file 16:
***In vivo*** **localization of** ***Nv*** **Lgl::mCherry in early cleavage embryo of** ***N. vectensis*** **development.** z-stack series corresponding to an early embryo co-expressing *Nv*Lgl::mCherry and Lifeact::mTq2 proteins (shown in Figure 8C, cleavage). Additional file 17:
***In vivo*** **localization of** ***Nv*** **Lgl::mCherry in late cleavage embryo of** ***N. vectensis*** **development.** z-stack series corresponding to a late embryo co-expressing *Nv*Lgl::mCherry and Lifeact::mTq2 proteins (shown in Figure 8C, cleavage). Additional file 18:
***In vivo*** **localization of** ***Nv*** **Lgl::mCherry in blastula stage of** ***N. vectensis*** **development.** z-stack series corresponding to a blastula stage embryo co-expressing *Nv*Lgl::mCherry and Lifeact::mTq2 proteins (shown in Figure 8C, blastula). Additional file 19:
***In vivo*** **localization of** ***Nv*** **Lgl::mCherry in gastrula stage of** ***N. vectensis*** **development.** z-stack series corresponding to a gastrula stage embryo co-expressing *Nv*Lgl::mCherry and Lifeact::mTq2 proteins (shown in Figure 8C, gastrula). Additional file 20:
**Immunostaining in** ***Nv*** **Lgl::mCherry injected embryos.** Gastrula of *Nv*Lgl::mCherry injected embryos was stained using a monoclonal antibody raised against mCherry (1:250; Clontech, Inc. 632543). Co-immunostaining with anti-*Nv*Lgl (A), anti-*Nv*Par-1 (B), anti-*Nv*aPKC (C), and anti-*Nv*Par-6 (D) confirm our observations made for each protein in fixed and live embryos: (A) and (B) demonstrate that *Nv*Lgl::mCherry, anti-*Nv*Lgl, and anti-NvPar-1 do in fact co-localize in the basolateral cortex of the cells. In addition, (C) and (D) show the complimentary localization of *Nv*Lgl::mCherry, *Nv*aPKC, and *Nv*Par-6. Additional file 21:
***In vivo*** **localization of** ***Nv*** **Par-3::mVenus in unfertilized egg of** ***N. vectensis*** **development.** z-stack series corresponding to an unfertilized egg co-expressing *Nv*Par-3::mVenus and Lifeact::mTq2 proteins (shown in Figure 9C, unfertilized). Additional file 22:
***In vivo*** **localization of** ***Nv*** **Par-3::mVenus in early cleavage embryo of** ***N. vectensis*** **development.** z-stack series corresponding to an early embryo co-expressing *Nv*Par-3::mVenus and Lifeact::mTq2 proteins (shown in Figure 9C, cleavage). Additional file 23:
***In vivo*** **localization of** ***Nv*** **Par-3::mVenus in late cleavage embryo of** ***N. vectensis*** **development.** z-stack series corresponding to a late embryo co-expressing *Nv*Par-3::mVenus and Lifeact::mTq2 proteins (shown in Figure 9C, cleavage). Additional file 24:
***In vivo*** **localization of** ***Nv*** **Par-3::mVenus in blastula stage of** ***N. vectensis*** **development.** z-stack series corresponding to a blastula stage embryo co-expressing *Nv*Par-3::mVenus and Lifeact::mTq2 proteins (shown in Figure 9C, blastula). Additional file 25:
***In vivo*** **localization of** ***Nv*** **Par-3::mVenus in gastrula stage of** ***N. vectensis*** **development.** z-stack series corresponding to a gastrula stage embryo co-expressing *Nv*Par-3::mVenus and Lifeact::mTq2 proteins (shown in Figure 9C, gastrula). Additional file 26:
***In vivo*** **localization of** ***Nv*** **Par-3::mVenus and** ***Nv*** **Lgl::mCherry at cellular level in gastrula stage of** ***N. vectensis*** **development.** Time series corresponding to a region of a gastrula stage embryo co-expressing *Nv*Par-3::mVenus, *Nv*Lgl::mCherry, and Lifeact::mTq2 proteins (shown in Figure 12C, gastrula). Arrowheads indicate the astral and centrosomal localization of *Nv*Par-3::mVenus. Images were taken with a 100× objective immersed in oil. Additional file 27:
***In vivo*** **localization of** ***Nv*** **Par-6::mVenus and** ***Nv*** **Lgl::mCherry in early cleavage embryo of** ***N. vectensis*** **development.** z-stack series corresponding to an early embryo co-expressing *Nv*Par-6::mVenus, *Nv*Lgl::mCherry and, Lifeact::mTq2 proteins (shown in Figure 10C, cleavage). Additional file 28:
***In vivo*** **localization of** ***Nv*** **Par-6::mVenus and** ***Nv*** **Lgl::mCherry in late cleavage embryo of** ***N. vectensis*** **development.** z-stack series corresponding to a late embryo co-expressing *Nv*Par-6::mVenus, *Nv*Lgl::mCherry, and Lifeact::mTq2 proteins (shown in Figure 10C, cleavage). Additional file 29:
***In vivo*** **localization of** ***Nv*** **Par-6::mVenus and** ***Nv*** **Lgl::mCherry in blastula stage of** ***N. vectensis*** **development.** z-stack series corresponding to a blastula stage embryo co-expressing *Nv*Par-6::mVenus, *Nv*Lgl::mCherry, and Lifeact::mTq2 proteins (shown in Figure 10C, blastula). Additional file 30:
***In vivo*** **localization of** ***Nv*** **Par-6::mVenus and** ***Nv*** **Lgl::mCherry in gastrula stage of** ***N. vectensis*** **development.** z-stack series corresponding to a gastrula stage embryo co-expressing *Nv*Par-6::mVenus, *Nv*Lgl::mCherry, and Lifeact::mTq2 proteins (shown in Figure 10C, gastrula). Additional file 31:
***In vivo*** **localization of** ***Nv*** **Par-3::mVenus and** ***Nv*** **Lgl::mCherry in early cleavage embryo of** ***N. vectensis*** **development.** z-stack series corresponding to an early embryo co-expressing *Nv*Par-3::mVenus, *Nv*Lgl::mCherry and, Lifeact::mTq2 proteins (shown in Figure 12C, cleavage). Additional file 32:
***In vivo*** **localization of** ***Nv*** **Par-3::mVenus and** ***Nv*** **Lgl::mCherry in late cleavage embryo of** ***N. vectensis*** **development.** z-stack series corresponding to a late embryo co-expressing *Nv*Par-6::mVenus, *Nv*Lgl::mCherry, and Lifeact::mTq2 proteins (shown in Figure 12C, cleavage). Additional file 33:
***In vivo*** **localization of** ***Nv*** **Par-3::mVenus and** ***Nv*** **Lgl::mCherry in blastula stage of** ***N. vectensis*** **development.** z-stack series corresponding to a blastula stage embryo co-expressing *Nv*Par-3::mVenus, *Nv*Lgl::mCherry, and Lifeact::mTq2 proteins (shown in Figure 12C, blastula). Additional file 34:
***In vivo*** **localization of** ***Nv*** **Par-3::mVenus and** ***Nv*** **Lgl::mCherry in gastrula stage of** ***N. vectensis*** **development.** z-stack series corresponding to a gastrula stage embryo co-expressing *Nv*Par-3::mVenus, *Nv*Lgl::mCherry, and Lifeact::mTq2 proteins (shown in Figure 12C, gastrula).

## Abbreviations

A/B: apico-basal
A/V: animal-vegetal
aPKC: atypical protein kinase C
hpf: hours post fertilization
Lgl: lethal giant larvae
Lifeact::mTq: Lifeact::mTurquoise2
MMM: Murray’s mounting media
*N. vectensis*: *Nv*, *Nematostella vectensis*
Par proteins: *Par*titioning-defective proteins
