# Supplementary figures and images for: Par system components are asymmetrically localized in ectodermal epithelia, but not during early development in the sea anemone Nematostella vectensis
Source: EvoDevo. 2015 May 9;6:20. doi: 10.1186/s13227-015-0014-6 (PMC4476184; doi:10.1186/s13227-015-0014-6)

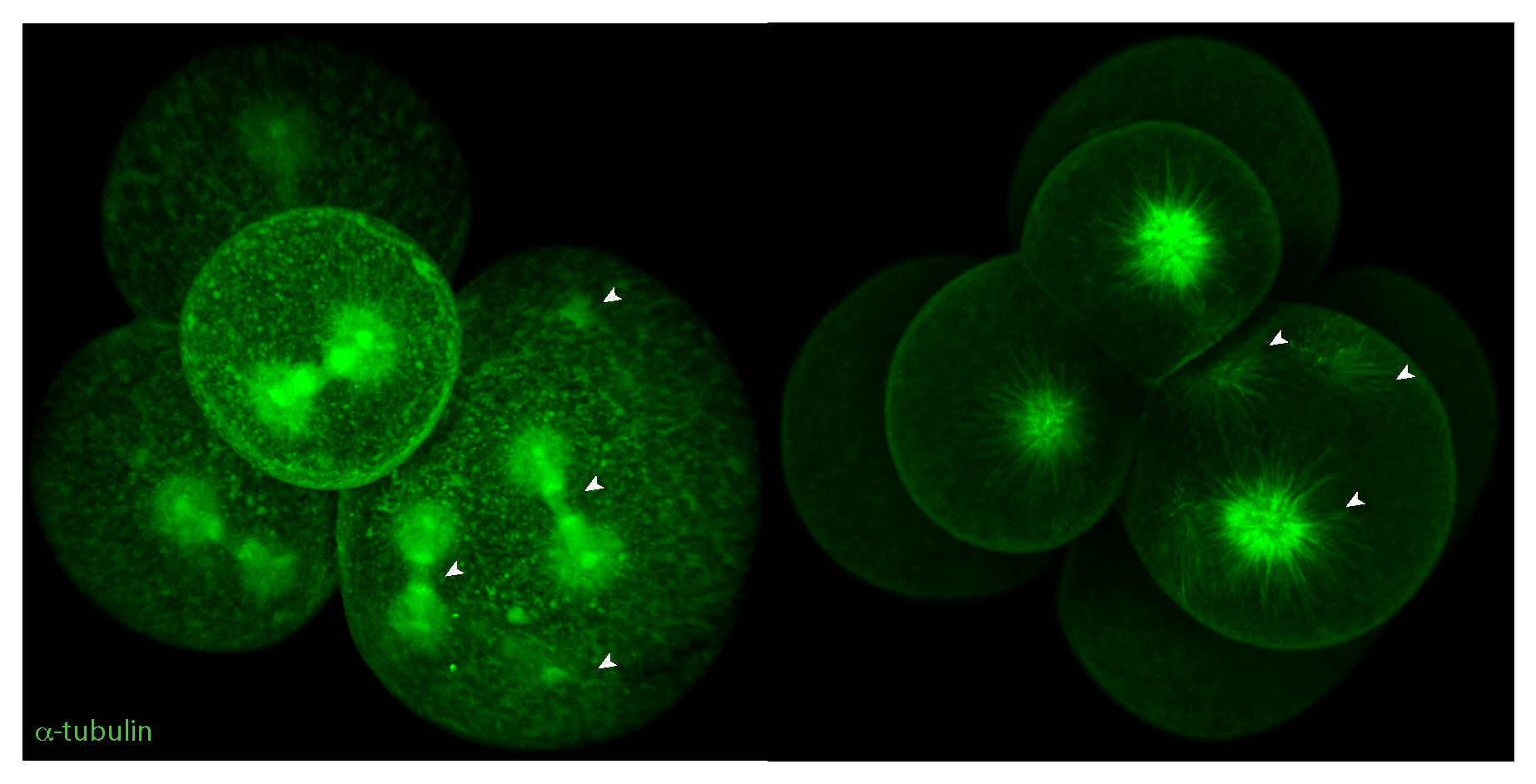

Supplement: Additional file 1: — Embryos of N. vectensis do not have distinguishable cell boundaries during cleavage stages. Immunostaining against alpha-tubulin labels more than one mitotic apparatus (arrowheads) enclosed by the same cell membrane. [file 13227_2015_14_MOESM1_ESM.png]

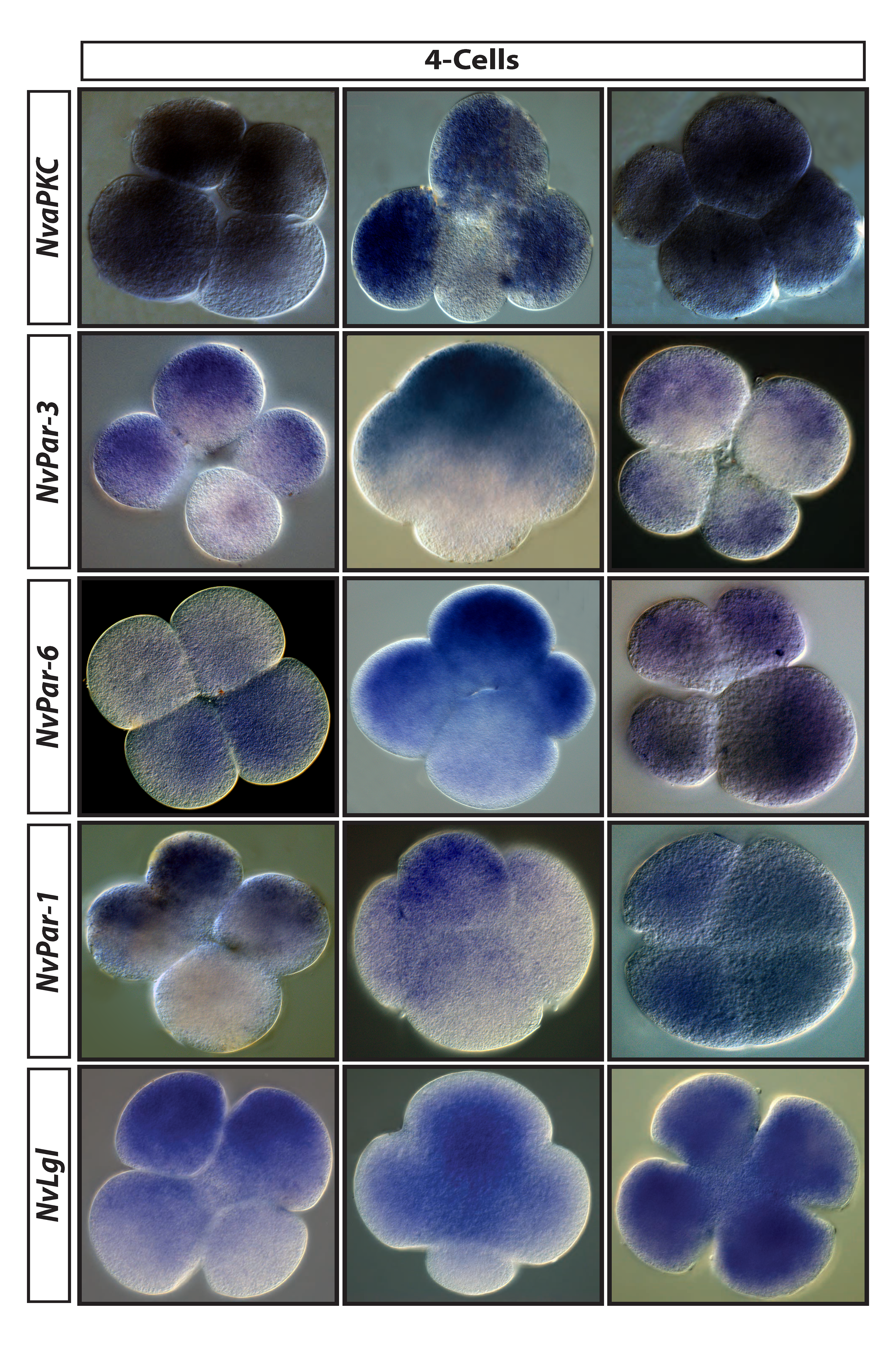

Supplement: Additional file 2: — Random distribution patterns of maternal mRNA at four-cell stages. Maternal mRNA of NvaPKC, NvPar-3, NvPar-6, NvPar-1, and NvLgl are randomly distributed during the four-cell stage of N. vectensis embryo (their pattern expression is not consistent). This suggests that mRNA asymmetric distribution in N. vectensis is not correlated with the A/V polarity. [file 13227_2015_14_MOESM2_ESM.png]

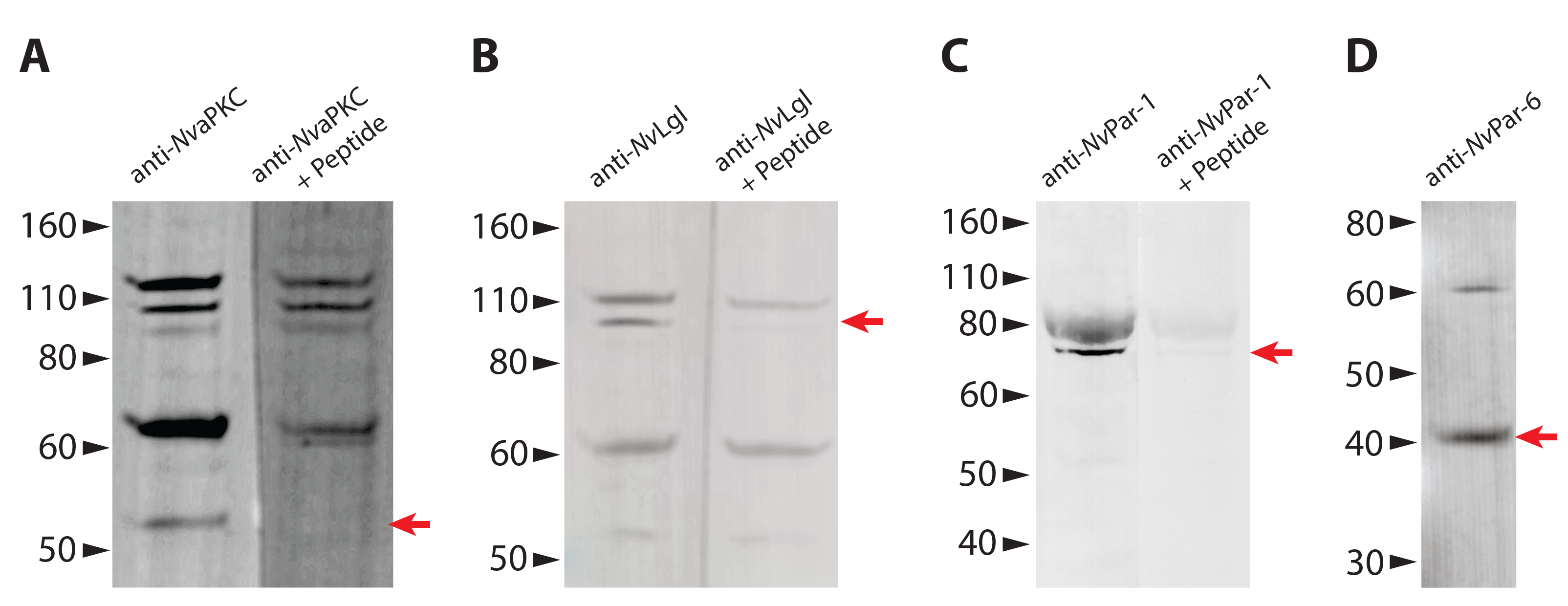

Supplement: Additional file 3: — Specificity of N. vectensis polyclonal affinity-purified antibodies by western blotting. Western blots of N. vectensis gastrula extracts using specific antibodies against NvaPKC (A), NvLgl (B), NvPar-6 (C), and NvPar-1 (D). A red arrow indicates the expected band of each protein. [file 13227_2015_14_MOESM3_ESM.png]

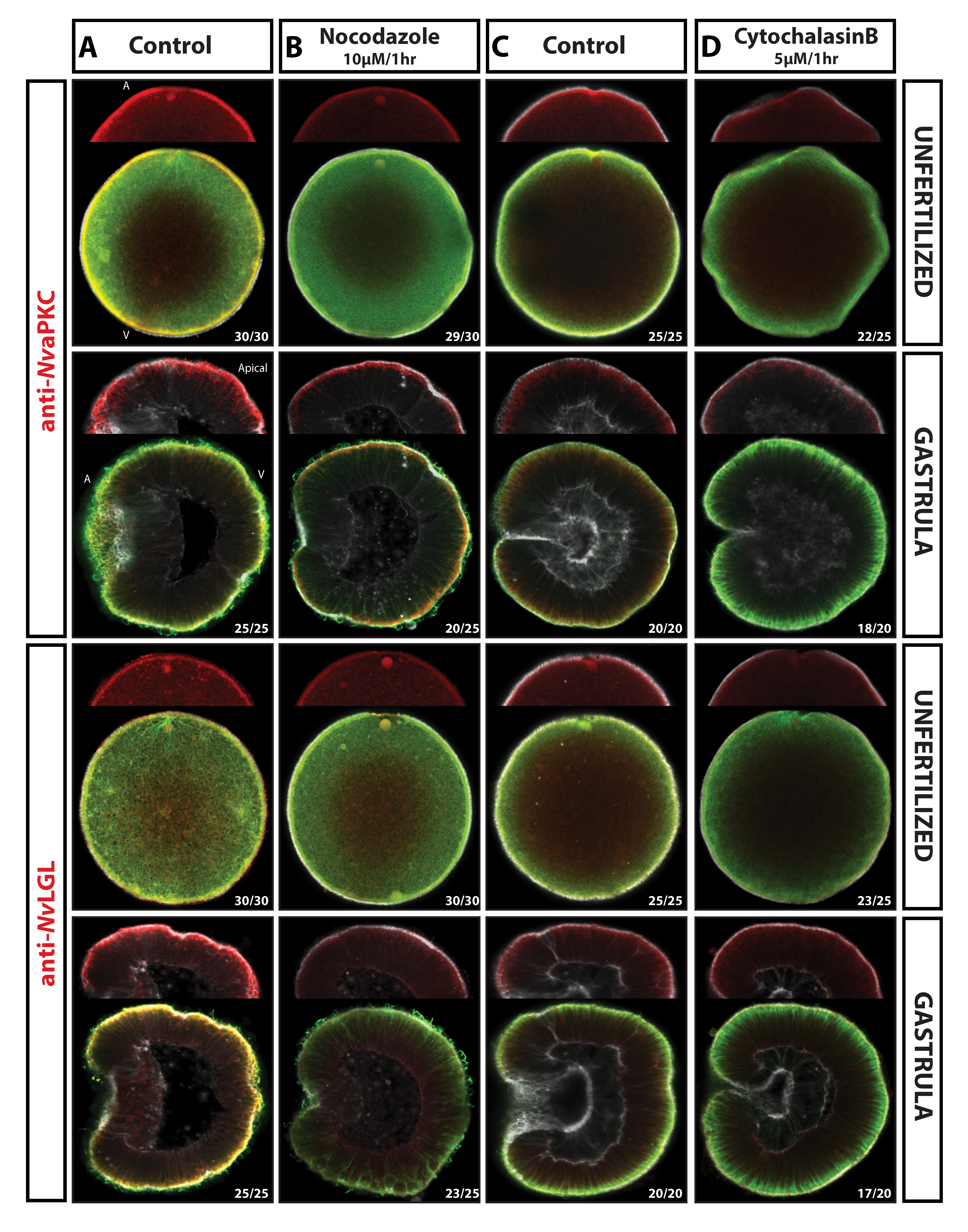

Supplement: Additional file 4: — The protein localization of Nv aPKC and Nv Lgl is associated with stable components of microtubule and actin cytoskeleton. Protein localization of NvaPKC and NvLgl is associated with stable components of microtubule and actin cytoskeleton. We tested the cytoskeletal dependency of intracellular localization by treating during 1-h embryos with drugs against the polymerization of microtubules (nocodazole 10 μM/1 h) and actin cytoskeleton (cytochalasinB 5 μM/1 h). During earlier stages, the localization of both NvLgl and NvaPKC is not affected by either treatment, with no clear polarization observed. Likewise, during gastrulation, the basolateral localization of NvLgl and the apical distribution of NvaPKC remain, suggesting that the localization of both proteins is associated with either stable microtubules or actin cytoskeleton. Colors green and gray are used to show tubulin and actin labeling, respectively. [file 13227_2015_14_MOESM4_ESM.png]

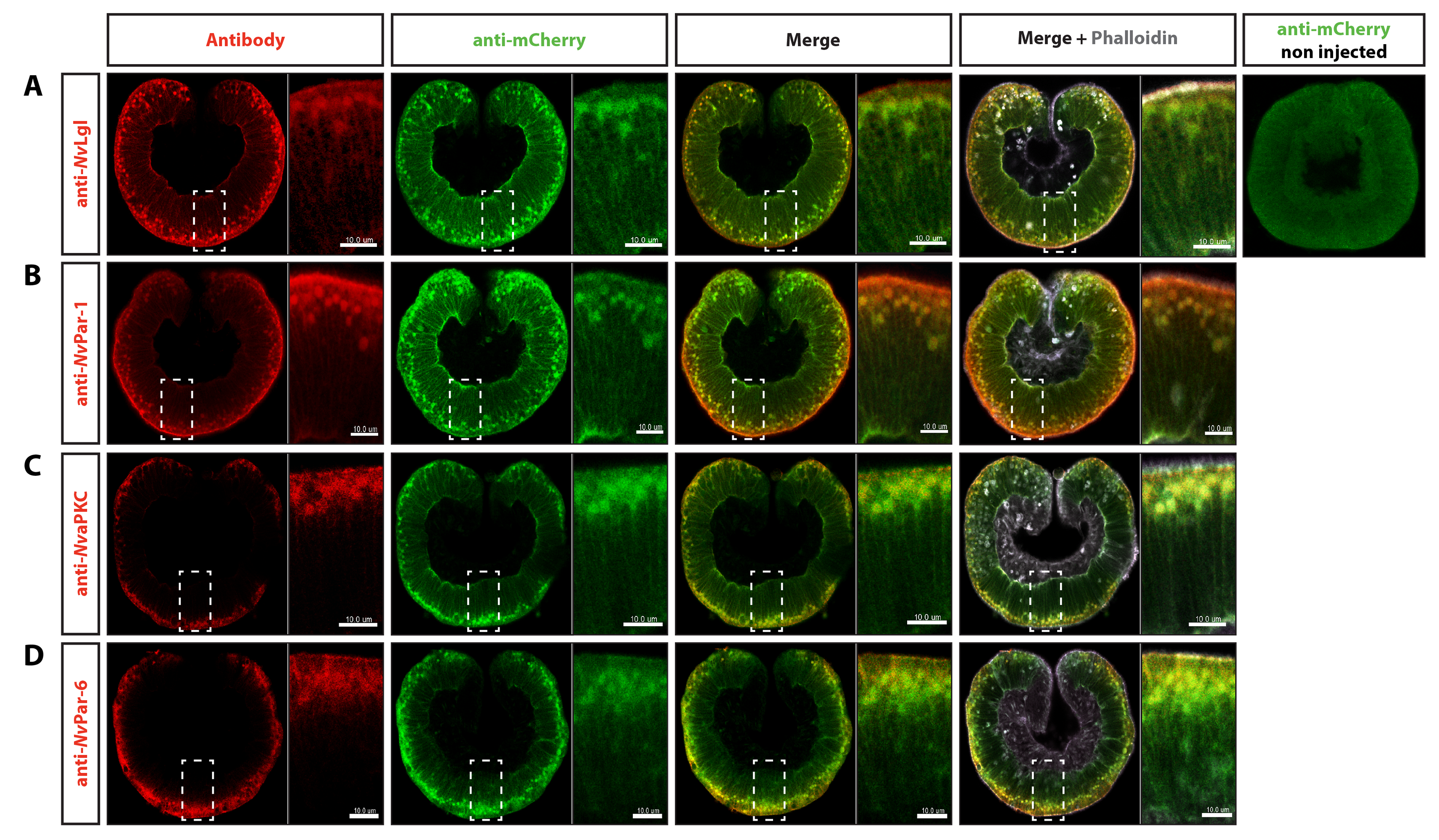

Supplement: Additional file 20: — Immunostaining in Nv Lgl::mCherry injected embryos. Gastrula of NvLgl::mCherry injected embryos was stained using a monoclonal antibody raised against mCherry (1:250; Clontech, Inc. 632543). Co-immunostaining with anti-NvLgl (A), anti-NvPar-1 (B), anti-NvaPKC (C), and anti-NvPar-6 (D) confirm our observations made for each protein in fixed and live embryos: (A) and (B) demonstrate that NvLgl::mCherry, anti-NvLgl, and anti-NvPar-1 do in fact co-localize in the basolateral cortex of the cells. In addition, (C) and (D) show the complimentary localization of NvLgl::mCherry, NvaPKC, and NvPar-6. [file 13227_2015_14_MOESM20_ESM.png]
